# Computer-Aided Prediction of the Interactions of Viral Proteases with Antiviral Drugs: Antiviral Potential of Broad-Spectrum Drugs

**DOI:** 10.3390/molecules29010225

**Published:** 2023-12-31

**Authors:** Pengxuan Ren, Shiwei Li, Shihang Wang, Xianglei Zhang, Fang Bai

**Affiliations:** 1 School of Life Science and Technology, Shanghai Institute for Advanced Immunochemical Studies, ShanghaiTech University, Shanghai 201210, China; renpx@shanghaitech.edu.cn (P.R.); lishw@shanghaitech.edu.cn (S.L.); wangshh12022@shanghaitech.edu.cn (S.W.); 2 School of Information Science and Technology, ShanghaiTech University, Shanghai 201210, China; 3 Shanghai Clinical Research and Trial Center, Shanghai 201210, China

**Keywords:** viral protease, 3CL^pro^, PAPs, inhibitor design strategies, drug repurposing

## Abstract

Human society is facing the threat of various viruses. Proteases are promising targets for the treatment of viral infections. In this study, we collected and profiled 170 protease sequences from 125 viruses that infect humans. Approximately 73 of them are viral 3-chymotrypsin-like proteases (3CL^pro^), and 11 are pepsin-like aspartic proteases (PAPs). Their sequences, structures, and substrate characteristics were carefully analyzed to identify their conserved nature for proposing a pan-3CL^pro^ or pan-PAPs inhibitor design strategy. To achieve this, we used computational prediction and modeling methods to predict the binding complex structures for those 73 3CL^pro^ with 4 protease inhibitors of SARS-CoV-2 and 11 protease inhibitors of HCV. Similarly, the complex structures for the 11 viral PAPs with 9 protease inhibitors of HIV were also obtained. The binding affinities between these compounds and proteins were also evaluated to assess their pan-protease inhibition via MM-GBSA. Based on the drugs targeting viral 3CL^pro^ and PAPs, repositioning of the active compounds identified several potential uses for these drug molecules. As a result, Compounds **1**–**2**, modified based on the structures of Ray1216 and Asunaprevir, indicate potential inhibition of DENV protease according to our computational simulation results. These studies offer ideas and insights for future research in the design of broad-spectrum antiviral drugs.

## 1. Introduction

The outbreak of Coronavirus Disease 2019 (COVID-19), caused by SARS-CoV-2, has had a significant impact on human health and lives [1,2]. In May 2023, the World Health Organization announced that the three-year epidemic is no longer considered a Public Health Emergency of International Concern (PHEIC) [3]. Although the most severe phase of the COVID-19 pandemic is over, people still have a long way to go to combat the virus-driven crisis. A lack of understanding of viruses underlines the global vulnerability to emerging diseases. The Global Virome Project report estimates that there are 1.67 million unknown viruses in mammalian and bird hosts [4]. Among these viruses, an estimated 650,000 to 840,000 can infect the human body and cause disease [4]. To date, only about 270 viruses are known to infect humans [1,2,4,5,6,7,8,9,10].

Since the 1960s, at least 95 small molecule inhibitors against viruses have been approved (Table S1) [11]. However, these small-molecule drugs only target 11 types of viruses (HSV, VZV, HCMV, VARV, HBV, HCV, SARS-CoV-2, FluV, RSV, HDV, and HIV), which account for only 4.07% of the 270 known viruses that can infect humans. In addition to targets related to viral DNA and RNA synthesis, e.g., DNA polymerase, RNA polymerase, and reverse transcriptase, proteases are also frequently targeted in the development of antiviral drugs. A few antiviral drugs targeting proteases such as 3CL^pro^ of SARS-CoV-2, NS3/4A of HCV, and the protease of HIV have been approved (details are below). The 3CL^pro^ of SARS-CoV-2, also known as the main protease (M^pro^), has the ability to cleave two polyproteins expressed by the virus genome into multiple non-structural functional proteins. This process plays a crucial role in virus translation and replication [12]. NS3/4A of HCV is embedded in the endoplasmic reticulum membrane of host cells [13,14]. Its primary function is similar to that of the 3CL^pro^ of SARS-CoV-2, which cleaves the polyproteins translated from the HCV genome into functional viral proteins [13,14]. The HIV protease cleaves the polyprotein Gag-Pol at multiple sites to produce mature viral components of HIV [15].

**Figure 1:**
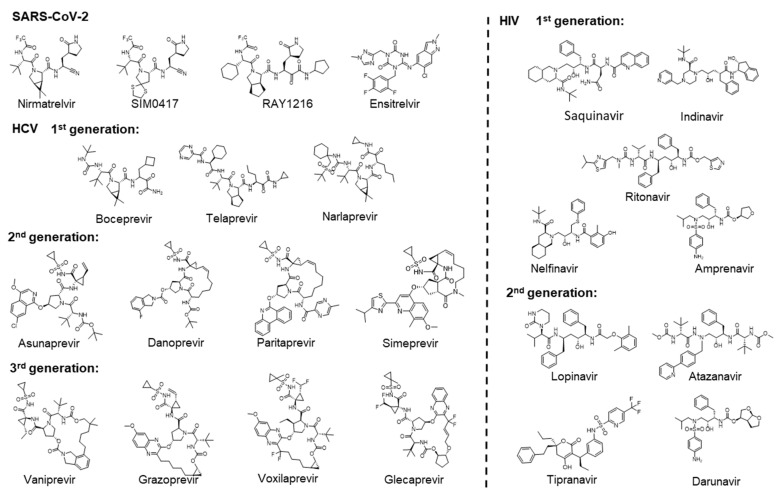
Two-dimensional chemical structures of approved drugs targeting SARS-CoV-2 3CL^pro^, HCV NS3/4A, and HIV proteases, respectively.

As shown in Figure 1, the approved drugs targeting 3CL^pro^ of SARS-CoV-2 are three covalent inhibitors: Nirmatrelvir [16,17], SIM0417 [18], and RAY1216 [19], as well as a non-covalent inhibitor, Ensitrelvir [20]. Nirmatrelvir and SIM0417 need to be combined with Ritonavir to increase the drug’s half-life in the human body. Three generations of drugs have been developed for HCV NS3/4A. Boceprevir [21], Telaprevir [22], and Narlaprevir [23] are first-generation Protease Inhibitors (PIs) that all contain covalent warheads (α, β-unsaturated aldehydes and ketones) capable of forming covalent bonds with the catalytic residue Ser. The second generation of PIs, including Asunaprevir [24], Danoprevir [25], Paritaprevir [26], and Simeprevir [27], do not contain functional groups to form covalent bonds and are equipped with hydrophobic aromatic groups P2, which significantly increases their inhibitory effect on proteases. The third generation of PIs, including Vaniprevir [28], Grazoprevir [29], Voxilaprevir [30], and Glecaprevir [31,32], have transformed the P1-P3 macrocycle of the second generation of PIs into a P2-P4 macrocycle. They have the advantage that they are effective against many genotypes, including a short treatment duration, infrequent oral administration, do not require the use of interferon in combination, and are not affected by multiple drug-resistant mutations. In the early 1980s, AIDS emerged as a new viral disease in the public eye, marking the beginning of a prolonged battle against the illness [33]. Two generations of drugs targeting HIV protease have been developed. Saquinavir is the first HIV protease inhibitor [34]. Subsequently, the other first-generation PIs of Ritonavir [35], Indinavir [36], Nelfinavir [37], and Amprenavir [38] were also approved. The second-generation PIs of Lopinavir [39], Atazanavir [40], Tipranavir [41], and Darunavir [42] are characterized by a greater emphasis on the pharmacokinetic properties, efficacy, and safety of the drugs. These drugs targeting HIV proteases all contain hydroxyl groups that form hydrogen bonds with the catalytic residue Asp. This interaction is crucial for inhibiting the activity of proteases. There are numerous other viral proteases that play crucial roles in the life cycle of viruses. For example, the 3CL^pro^ of viruses in the order Picornavirales, such as FMDV [43], EV-A71 [44], HRV [45], and NV [46], and the proteases of viruses in the order Amarillovirales, such as DENV [47], could cleave the viral polyproteins. Based on the design process of approved drugs, we can develop drugs for similar targets.

Understanding the development process and the mechanisms of antiviral drugs is important for designing more efficient drugs. In this article, we have collected protease sequences from viruses that infect humans and depicted the protease profiles to illustrate the distribution of viral proteases among viruses. We focused on two types of proteases, 3CL^pro^ and PAPs, analyzed their sequences, structures, and substrate features, and performed binding predictions for these proteins and some approved anti-virus drugs. The initial binding poses for drugs-proteins were constructed by referring to the experimentally determined binding complexes of the protease and ligands. The studied drugs were positioned appropriately through structure alignment, and the residues around the small molecules were treated as flexible in order to refine the complex structures.

## 2. Results

### 2.1. The Protease Profiles of Human-Infective Viruses

**Figure 2:**
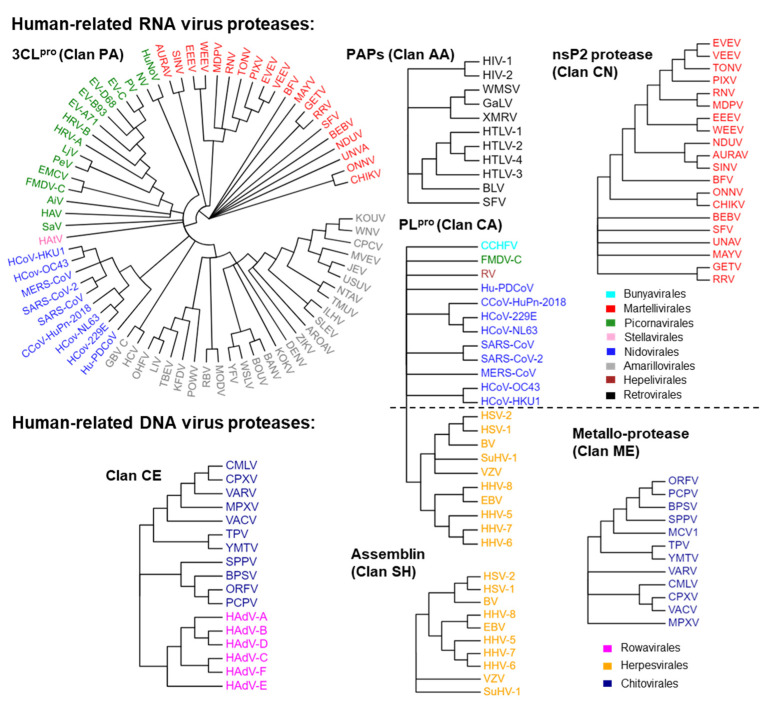
The protease profiles of human-infective viruses. Proteases in the same group of clans are shown in one tree. Viruses in the same order are marked with the same colors.

We initially analyzed which viruses related to humans are capable of expressing proteases and the types of proteases that can be expressed. There are approximately 270 known viruses that infect humans [1,2,4,5,6,7,8,9,48]. According to the order classification, these viruses include Renovirales and Durnavirales of the dsRNA virus class; Monongavirales, Bunyavirales, and Articulaviales of the (−) ssRNA virus class; Picornavirales, Nidovirales, Amarillovivirales, Martellivirales, Hepelivirales, and Stellavvirales of the (+) ssRNA virus class; Retrovirales of the ssRNA (RT) virus class; Piccovirales and Aneloviridae (family) of the ssDNA virus class; Rowavirales, Herpesvirales, Zurhausenvirales, Sepolyvirales, and Chitovirres of the dsDNA virus class; Hepadnaviridae of the dsRNA (RT) virus class. Among these viruses, those capable of expressing proteases are mainly distributed in six orders of the (+) ssRNA virus class, Retrovirales of the ssRNA (RT) virus class, Bunyavirales of (−) ssRNA virus class, and Rowavirales, Herpesvirales, and Chitovirres of the dsDNA virus class. Based on this classification, we extracted and collected the protease sequences of these viruses and generated protease profiles of human-infective viruses to visualize the distribution of these targets on the constructed evolutionary trees (Figure 2). These proteases are classified into seven clans (Table 1), including a Cys/Ser protease (clan PA), three Cys proteases (clan CA, clan CN, and clan CE), a Ser protease (clan SH), an Asp protease (clan AA), and a Metallo-protease (clan ME). The same clan has the same fold. The proteins in the group of clan PA are 3-chymotrypsin-like proteases (3CL^pro^). Proteins in the group of clan AA are pepsin-like aspartic proteases (PAPs). The proteins in the group of clan CA are papain-like proteases (PL^pro^).

As shown in Table 1, we have summarized the catalytic residues and functions of these proteases. Except for HEV and RV of Hepelivirales, other (+) ssRNA viruses can express 3CL^pro^. The capsid of alphaviruses (order Martellivirales) exhibits 3CL^pro^ activity, which is utilized for self-cleavage [49,50,51]. The capsid of an alphavirus is released from the polyprotein through a one-step enzymatic cleavage, after which its protease activity is inactivated [49,50,51]. The 3CL^pro^ of Picornavirales, Nidovirales, Amarillovirales, and Stellavirales is used to cleave polyproteins that are expressed in the viral genome and are not components of the viral particles [12,13,14,52,53]. Alphavirus cleaves the polyprotein using nsP2 proteins (clan CE) [54].

Several viruses can produce proteins with PL^pro^ activities. The PL^pro^ of the coronavirus in Nidovirales proteases, in addition to 3CL^pro^, have multiple cleavage sites on the viral polyprotein and act as deubiquitinases (DUBs) [55,56,57]. HCoV-OC43, HCoV-HKU1, HCoV-229E, HCoV-NL63, and CCoV-HuPn-2018 express two PL^pro^ (PLP1 and PLP2) [58]. PLP2 has the same function as PL^pro^ in other coronaviruses [55,56,57]. PLP1 and PLP2 collaborate to promote virus replication. L^pro^ (the leader protein) is the first protein encoded on the FMDV (Picornavirales) polyprotein and belongs to the PL^pro^ type. It self-cleaves from the precursor of the polyprotein, cleaves the host translation initiation factor eIF-4G, leading to a decrease in host cap-dependent mRNA translation, and also exhibits DUBs activity [56]. The PL^pro^, expressed by RV (Hepelivirales), cleaves viral polyproteins into nonstructural proteins [56]. PL^pro^, expressed by RV (Hepelivirales), cleaves viral polyproteins into nonstructural proteins [59]. The large tegument protein of Herpesvirales and the large (L) protein of CCHFV (Bunyavirales) are both components of the viral particle and contain a PL^pro^ domain, which has been shown to have DUB activity [56,60].

The proteases of the assemblins (clan SH) of herpesviruses (Herpesvirales) are crucial for the formation of the nucleocapsid of the virus and virus replication. During the viral assembly process, the natural substrates of the assemblins are the viral protease precursor and the viral-assembly protein [61,62].

In the family of Retroviridae, 11 human-infectable viruses, including HIV-1, HIV-2, WMSV, GaLV, XMRV, SFV, BLV, HTLV-1, HTLV-2, HTLV-3, and HTLV-4, express PAPs (clan AA) to cleave viral polyproteins. These PAPs are also structural proteins of the viruses that are distributed in the viral matrix. The protease of SFV is located on the same protein as the reverse transcriptase and ribonuclease H [63].

The core protease (I7) of the Poxviridae (Chitovirales) and adenain (the adenovirus endoprotease) of the Adenoviridae (Rowavirales) belong to the group of clan CE. The core protease is necessary for cleaving the viral membrane and core proteins during virus assembly [64,65]. Adenain, which is responsible for the uncoating and maturation of virions, converts immature viral particles into mature viral particles with infectious ability and releases them from infected cells [66]. In addition, poxviridae also express a Metallo-protease (clan ME), which plays a role in the maturation of viral proteins [67].

The classification of viral proteases is important for developing broad-spectrum antiviral inhibitors, establishing precise antiviral treatment systems, and increasing the global stock of antiviral drugs.

### 2.2. 3-Chymotrypsin-Like Proteases

#### 2.2.1. The Sequences, Structures, and Substrates of the Class of 3CL^pro^

**Figure 3:**
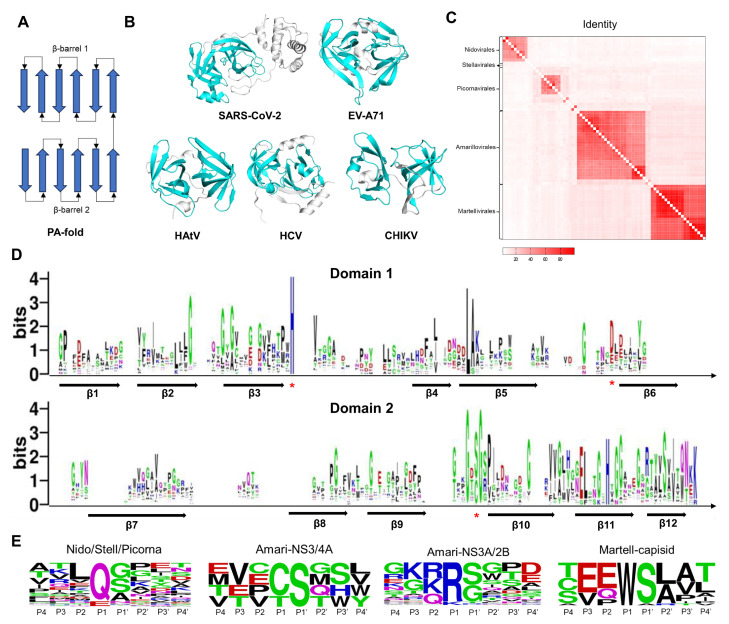
The folding patterns, sequences, structures, and substrates of viral 3CL^pro^. (**A**) General folding topology diagram for the class of 3CL^pro^. Arrows represent β-sheets. (**B**) The 3D structures of SARS-CoV-2, EV-A71, HAtV, HCV, and CHIKV 3CL^pro^. The proteins are represented as cartoon models. The catalytic domains are colored in cyan. (**C**) The heat map shows the percentage of sequence identity for 3CL^pro^ proteins between different viruses. (**D**) Sequence logo of multiple sequence alignment. Arrows represent β-sheets. Asterisks represent catalytic residues. (**E**) Sequence logo of 3CL^pro^ cleavage sites. Residues are scaled according to their frequencies at each position.

Chymotrypsin comprises two β-barrel-like domains (Figure 3A). Each β-barrel consists of 6 β-Sheets. The catalytic active center is located between two domains. As shown in Figure 3B, we present the 3CL^pro^ structures of typical viruses from five viral orders. The catalytic domains of SARS-CoV-2, EV-A71, HAtV, HCV, and CHIKV 3CL^pro^ exhibit the same structural fold as chymotrypsin. The active center of Coronaviridae 3CL^pro^ consists of the catalytic dyad His-Cys. The active center of Picornavirales 3CL^pro^ consists of the catalytic triad His-Asp/Glu-Cys. The active center of astrovirus Flaviviridae and alphavirus 3CL^pro^ consists of the catalytic triad His-Asp/Glu-Ser.

A total of 73 sequences of viral 3CL^pro^ proteins were collected (Figure 2). We calculated the sequence identities between these proteins and illustrated the matrix diagram in Figure 3C. Identity values can be found in the Supplementary Materials. Conserved sequences are concentrated in the same order. We clustered these proteins into 13 classes based on identity values. Sequences with identity values greater than 30% were grouped into one class. The identity between the 3CL^pro^ of coronaviruses is greater than 30% (class 1). HAtV 3CL^pro^ of Stellavvirales was classified as class 2. For the viruses in the order of Picornavirales, we classified these viruses into 8 classes: class 3 (EV-A71, EV-B93, EV-D68, EV-C, PV, HRV-B, and HRV-A), class 4 (PeV and LjV), class 5 (NV and HuNoV), class 6 (EMCV), class 7 (AiV), class 8 (FMDV), class 9 (HAV), and class 10 (SaV). In the Flaviviridae family, the proteases of other 25 viruses, such as ZIKV, JEV, and DENV, belong to the NS3/2B (class 11), while the proteases of HCV and HGV belong to the NS3/4A (class 12). NS4A and NS2B are both cofactors of proteases. The identity values between the 3CL^pro^ of alphaviruses are all greater than 30% (class 13). We performed sequence alignment with structural constraints to align the 3CL^pro^ sequences and depicted a sequence logo for the two domains. As shown in Figure 3D and Figure S1, there are 6 β-sheets distributed in each of the domains. Each β-sheet is arranged in the same order. The positions of the catalytic residues His and Cys/Ser are highly conserved.

Meanwhile, we also collected the substrates of these proteases. The cleavage sites of some viral proteases are not annotated, but they share similarities with homologous sequences in terms of cleavage sites and substrate sequences. Then, we created sequence logo diagrams for the substrate sequences of the 13 classes of viruses (Figure S2). Due to the conservative P1 residues, we further divided the substrate sequence into four categories based on the P1 position. The sequence logo diagram is shown in Figure 3E. Substrates of Coronaviridae, Picornavirales, and HAtV 3CL^pro^ have a high proportion of glutamine (Q) or glutamic acid (E) at the P1 position and were classified into a single category. The substrates of Flaviviridae NS3/2B have a high proportion of Arginine (R) or Lysine (K) at the P1 position and were classified as a single type. Substrates of the Flaviviridae NS3/4A protease have a high proportion of Cysteine (C) or Threonine (T) at the P1 position and were classified as a single type. Substrates of Alphavirus capsid have a high proportion of Tryptophan (W) at the P1 position and were classified as one type. The substrate sequence preference indicates the binding preference of protein sites. In Figure S3A, the S1 position of the protein pocket corresponds to the P1 residue. By combining the residues at the S1 position with reported complex structures, we can summarize the patterns of the functional groups of inhibitors at the S1 site (Figure S3B). The high proportion of Q/E at the P1 position in Coronaviridae, Picornavirales, and HAtV is attributed to the presence of the Histidine (His) residue at the S1 position, which acts as a hydrogen bond donor. We offer various fragments for designing inhibitors, including amide groups, lactam rings, carboxyl groups, and nitrogen heterocycles. Due to the presence of Aspartic acid (Asp) in Flaviviridae NS3/2B at the S1 site, positively charged groups are suitable for this position, such as guanidine and amino groups. Hydrophobic groups are suitable for the S1 site of Flaviviridae NS3/4A. A Tryptophan (Trp) residue inserts into the S1 site of the alphavirus capsid. In addition, hydrophobic groups are suitable for the S2 site of Coronaviridae 3CL^pro^, such as the P2 position of Nirmatrelvir, SIM0417, RAY1216, and Ensitrelvir. For the S2 site of Flaviviridae NS3/2B, positively charged groups are suitable for this position, such as guanidine and amino groups.

The analysis and comparison of the sequence, structure, and substrate characteristics of viral 3CL^pro^ are highly significant for designing inhibitors targeting such targets.

#### 2.2.2. The Drug-Protein Complex Structure Prediction and Modeling Method

**Figure 4:**
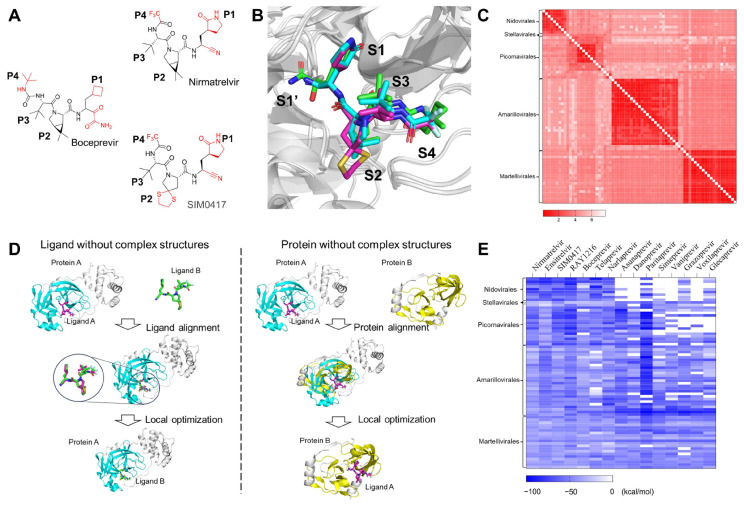
Computational prediction and analysis of the ligand-protein binding complex structures. (**A**) The 2D chemical structures of Boceprevir, Nirmatrelvir, and SIM0417. (**B**) The superimposition of HCV NS3/4A with Boceprevir (PDB code: 3LOX) and SARS-CoV-2 3CL^pro^ with Nirmatrelvir (PDB code: 7RFS) and SIM0417 (PDB code: 8IGX). Boceprevir (green), Nirmatrelvir (cyan), and SIM0417 (magenta) are shown as sticks. The proteins are shown as cartoon models. (**C**) The heat map shows the RMSD for those viral 3CL^pro^ proteins. (**D**) The pipeline of the computational ligand-protein complex structure prediction and optimization strategy. (**E**) The heat map shows the predicted binding affinities (measured using MM-GBSA) for the 15 studied SARS-CoV-2 or HCV drugs against the 73 3CL^pro^ proteins of different viruses.

If the two protein sequences are significantly different, i.e., the identity is lower than 30%, the two structures cannot be aligned using sequence-based 3D structural alignment methods. Sequence-independent alignment enables researchers to visually identify similarities and differences between proteins from a 3D perspective. The structures of Nirmatrelvir and SIM0417 closely resemble those of Boceprevir (Figure 4A). The identity between HCV NS3/4A and SARS-CoV-2 3CL^pro^ is only 4.87%. We superimposed the 3D complex structures of SARS-CoV-2 3CL^pro^-Nirmatrelvir and SARS-CoV-2 3CL^pro^-SIM0417 on the complex structure of HCV NS3/4A-Boceprevir using cealign [68,69], a sequence-independent alignment method (Figure 4B). The root mean square deviations (RMSD) are 4.395 Å and 4.901 Å, respectively, and the P1-P4 segments of these molecules have matching 3D structures. The above results suggest that the inhibitors may have similar binding modes when the proteins have highly conserved active sites. In molecular docking, small molecules are flexible, while proteins are rigid or semi-flexible. This method cannot accurately predict the binding mode between small molecules and homologous proteins with significant differences. Constructing complex structures through structural alignment and local refinement is suitable for this situation.

Of the 73 sequences that we have collected, the structures of 34 sequences have already been determined experimentally. Approximately 35 sequences have no experimental structures, but they have templates with an identity of more than 30% in the PDB database. Four sequences have neither structures nor templates with similar sequences. The emergence of AlphaFold enables researchers to accurately predict protein structures [70]. ColabFold simplifies and accelerates the prediction of the 3D structure of proteins [71]. We used ColabFold to predict the 3D structures of sequences without experimental structures, superimposed these structures, and calculated the RMSD between them (Figure 4C). The RMSD of these 3D structures ranges from 0.122 Å to 7.611 Å. The structural comparison results indicate that there may be slight spatial differences in the positions of protease pockets S1, S2, S3, and S4, but the overall arrangement remains consistent.

The computational binding complex prediction strategy is based on the hypothesis that a ligand will bind to the same binding site with similar binding poses while it interacts with similar proteins. As shown in Figure 4D, the main idea is to build the complex structures of target proteins and ligands according to the known experimental complex structures. For small molecules without experimental complex structures, we aligned them to the experimentally determined bound conformations and the binding positions of the ligands, which are structurally similar, by using Ligand Alignment in the Schrödinger suite to generate initial binding poses for them, then merged these conformations with the target protein structures to create complex structures, and performed substantial local optimization to obtain the final complex structures. For proteins without experimental complex structures, we superimposed their structures on proteins with reported complex structures, extracted ligands from the complex structures, merged these conformations, and conducted optimization in the local region of the binding sites to build the complex structures. The approved drugs have been tested for their bioactivities, pharmacokinetics, and toxicities before being administered to the human body. Repositioning such drugs can expedite the development of inhibitors. We constructed complex structures of 4 protease inhibitors of SARS-CoV-2 and 11 protease inhibitors of HCV against 73 viral 3CL^pro^ proteins using this method and then estimated the binding affinities via the method of molecular mechanics Poisson–Boltzmann surface area (MM-GBSA) [72]. The binding energy matrix is shown in Figure 4E. The concrete application of this strategy will be introduced as follows:

#### 2.2.3. Drug Repurposing

Drug repurposing is a strategy that aims to find alternative uses for approved or investigational drugs [73]. This strategy allows researchers to take advantage of testing drugs in the human body, shortening drug development time and saving social resources. From the binding energy we calculated (Figure 4E), 4 protease inhibitors targeting SARS-CoV-2 and 11 protease inhibitors targeting HCV can inhibit the 3CL^pro^ of various viruses. The Δ*G* values of binding affinities calculated between Coronaviridae 3CL^pro^ and Nirmatrelvir, Ensitrelvir, SIM0417, and RAY1216 are all below −40 kcal/mol. Four SARS-CoV-2 inhibitors also exhibited strong binding with Picornavirales 3CL^pro^, such as EMCV (−52.37 kcal/mol, RAY1216), AiV (−63.51 kcal/mol, RAY1216), FMDV (−81.61 kcal/mol, RAY1216), EV-A71 (−69.62 kcal/mol, SIM0417), HAV (−66.78 kcal/mol, RAY1216), PeV (−82.22 kcal/mol, RAY1216), SaV (−73.96 kcal/mol, RAY1216), NV (−60.88 kcal/mol, Nirmatrelvir), and so on. For Flaviviridae and alphavirus 3CL^pro^, they also demonstrate strong binding with certain proteins, such as OHFV (−72.57 kcal/mol, RAY1216), GBV (−61.77 kcal/mol, RAY1216), and VEEV (−61.04 kcal/mol, RAY1216). However, due to the differences in the substrate sequences, we believe that these molecules are more suitable as reference molecules for Flaviviridae and alphavirus 3CL^pro^. The structures of Boceprevir, Telaprevir, and Narlaprevir are similar to those of Nirmatrelvir, SIM0417, and RAY1216. These compounds could have potential for inhibiting various viruses. Other inhibitors of HCV NS3/4A exhibit weak bindings to various viruses because of the steric hindrance introduced by their macrocyclic or large P2 groups (Figure S4).

**Figure 5:**
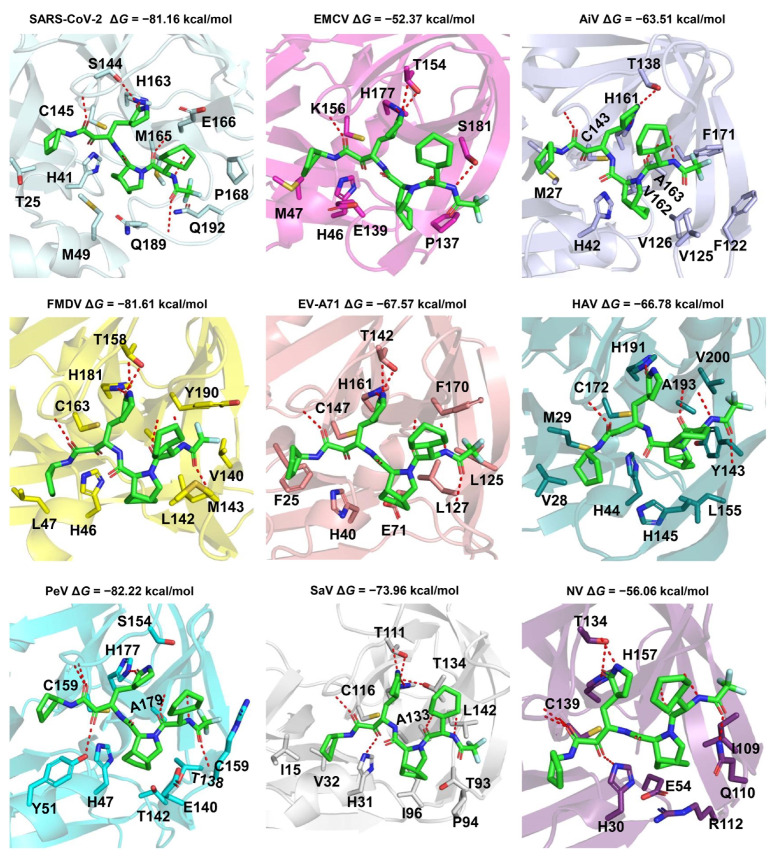
Predicted binding modes of Ray1216 against the 3CL^pro^ proteins of Picornavirales. The proteins are shown as cartoon models. The Ray1216 and interaction residues are depicted as sticks. The red dashed lines are hydrogen bonds between the ligand and protein. Picornavirales have the viruses EMCV, AiV, FMDV, EV-A71, HAV, PeV, SaV, and NV. The binding mode for Ray1216 and the 3CL^pro^ of SARS-CoV-2 is used as a control (PDB code: 8IGN) [19]. The PDB files of FMDV (PDB code: 2WV5), EV-A71 (PDB code: 3SJO), HAV (PDB code: 2HAL), and NV (PDB code: 5T6D) 3CL^pro^ are retrieved from the PDB database [74,75,76,77] and used in this study. The other protein structures were predicted by ColabFold [71].

Energy is one of the indicators of the binding affinity between a ligand and a protein pair. The binding mode of a ligand-protein can provide insight into molecular interactions and is crucial for drug design. RAY1216 exhibits strong binding energy with multiple 3CL^pro^ viruses. We predicted the binding modes of the typical 3CL^pro^ viruses from thirteen different classes of viruses (Figure 5 and Figure S5). The binding modes between RAY1216 and eight 3CL^pro^ proteins of viruses in the order Picornavirale (EMCV, AiV, FMDV, EV-A71, HAV, PeV, SaV, and NV) are similar to the binding mode between RAY1216 and 3CL^pro^ of SARS-CoV-2 (PDB code: 8IGN). According to our predicted binding complex structures, RAY1216 was observed to be able to fit snugly into the active pockets of these above-mentioned 3CL^pro^ proteins and form hydrogen bond interactions with the conserved residue His in the S1 pocket. The α, β-unsaturated aldehyde and ketone of RAY1216 are in close proximity to the catalytic residue cysteine and have the potential to form covalent bonds. It also forms hydrogen bonds with the main chain of some residues around it. It is noteworthy that the 3CL^pro^ of AiV, PeV, and SaV do not have experimentally determined structures, nor do they have templates with similar sequences. For HAtV, the binding mode suggests a potential interaction between Ray1216 and H566 of S1. However, the presence of N569 may contribute to an additional hydrogen bond with the ligand, leading to an observed lower binding energy between Ray1216 and HAtV 3CL^pro^ (Figure S5). RAY1216 also exhibits strong binding with DENV NS3/2B, HCV NS3/4A, and CHIKV capsid. Further modifications are required for Ray1216 in order to accommodate the preference of S1 and S2 pockets for DENV NS3/2B and HCV NS3/4A. The self-cleaves, and the C-terminal residue Trp occupies the active site after cleavage (Figure S3A). Whether this site is suitable for designing inhibitors requires further study.

The above results indicate that Ray1216 has therapeutic potential for various Picornavirales, and can be treated as a lead inhibitor for Flaviviridae and alphaviruses.

#### 2.2.4. Proposal for a Drug Optimization Strategy

**Figure 6:**
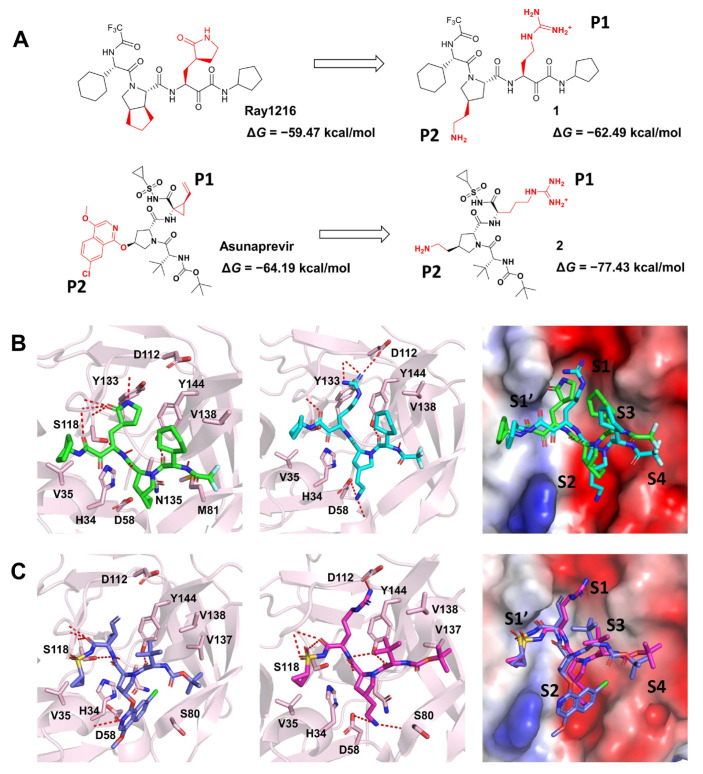
Inhibitors proposed against DENV protease are based on Ray1216 and Asunaprevir. (**A**) Schematic representation of the inhibitor design of DENV protease. (**B**) The binding modes of DENV NS3/2B with Ray1216 and compound **1**. (**C**) The binding modes of DENV NS3/2B with Asunaprevir and compound **2**. DENV proteases are represented as cartoon models and the surface of the electrostatic potential. Ray1216 (green), compound **1** (cyan), Asunaprevir (blue), compound **2** (magenta), and interaction residues are shown as sticks.

We can thus develop inhibitors with a broad spectrum of activity for virus targets with similar sequences. For targets with significant differences in sequences but similar folding and structures, we can design and modify inhibitors based on existing viral drugs. Till now, there have been no specific drugs for Dengue, caused by the dengue virus (DENV). Hence, we propose some modified molecules, starting with Ray1216 and Asunaprevir for DENV protease (NS3/2B). Due to the preference of the S1 and S2 sites or substrate P1 and P2 characteristics, we replaced the P1 and P2 groups of Ray1216 and Asunaprevir with guanidine and amino groups (Figure 6A). The complex structures of DENV protease with compounds **1** and **2** were constructed, and the Δ*G* was calculated by the above method. Δ*G* between compound **1** and DENV NS3/2B (−62.49 kcal/mol) was lower than Δ*G* between Ray1216 and DENV NS3/2B (−59.47 kcal/mol), indicating a stronger binding. Δ*G* between compound **2** and DENV NS3/2B (−77.43 kcal/mol) was lower than that between Asunaprevir and DENV NS3/2B (−64.19 kcal/mol). As shown in Figure 6B,C, the guanidino groups of compounds **1**–**2** are more suitable for binding to the S1 site compared to the γ-lactam group of Ray1216 or the alkene group of Asunaprevir. The electrostatic potential surface of proteins also displays that electronegative S1 and S2 sites pair with electropositive guanidine and amino groups. Taken together, the results indicate that compounds **1**–**2** we designed may have potential anti-DENV activities.

### 2.3. Pepsin-Like Aspartic Proteases

**Figure 7:**
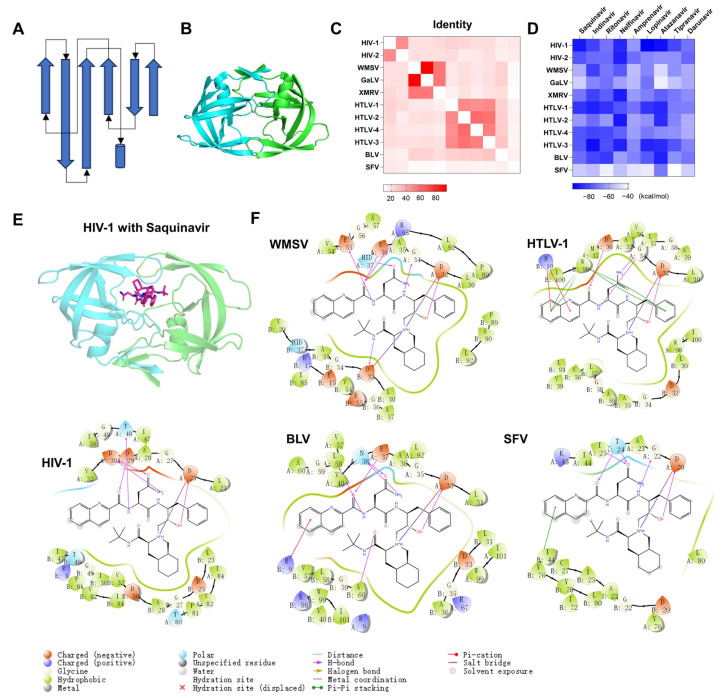
The predicted binding complexes of HIV protease inhibitors against PAPs of Retrovirales. (**A**) Topology diagram of PAPs. Arrows represent β-sheets. Cylinders represent α helices. (**B**) The 3D structure of HIV PAPs. The proteins are represented as cartoon models. The catalytic domains are colored cyan and green, respectively. (**C**) The heat map shows the percentage of sequence identity for PAPs proteins between different viruses. (**D**) The heat map shows the predicted binding affinities (measured using MM-GBSA) for the nine HIV drugs against the 11 PAPs proteins of different viruses. (**E**) The complex structure and binding mode of HIV-1 PAPs with Saquinavir. Saquinavir is depicted as a stick. (**F**) The binding modes between WMSV, HTLV-1, BLV, or SFV PAPs and Saquinavir.

Pepsin-like aspartic proteases (PAPs) include two domains with similar folding. Each domain is composed of multiple stranded β-sheets and a few helices (Figure 7A). Retroviral PAPs consist of two identical catalytic subunits (Figure 7B). The catalytic site is located between two subunits. Each subunit has a catalytic residue (Asp) in the same position. We collected the sequences of 11 Retrovirales proteases and calculated their identities (Figure 7C). Classification was performed according to a cutoff of identity > 30%. The Retrovirales PAPs include five classes: class 1 (HIV-1 and HIV-2), class 2 (WMSV, GaLV, and XMRV), class 3 (HTLV-1, HTLV-2, HTLV-3, and HTLV-4), class 4 (BLV), and class 5 (SFV). We performed sequence alignment for 11 PAPs (Figure S6). The sequence logo of alignment is shown in Figure S7A. β-sheets and α-helics are arranged in order. The positions of the catalytic residue Asp are very conservative. We also collected substrates for the PAPs (Figure S7B). The substrate-cleaving characteristics of SFV protease are uncertain. The cleaving features of other viral proteases are similar. The P1 residue is often a hydrophobic residue. There are six PAPs without experimental report structures. We predicted the dimer structures with ColabFold and superimposed the 3D structures of 11 PAPs. The RMSD matrix diagram is shown in Figure S7C. The RMSD ranges from 0.787 to 6.221 Å.

We constructed complex structures of 11 PAPs with 9 HIV protease inhibitors through a local optimization strategy and depicted the Δ*G* matric diagram between ligands and proteins (Figure 7D). Δ*G* are all less than −39.33 kcal/mol. We took Saquinavir, for example, to demonstrate the binding modes of HIV-1, WMSV, HTLV-1, BLV, and SFV PAPs with saquinavir. The complex structures of WMSV, HTLV-1, BLV, and SFV PAPs with saquinavir are similar to the complex structure of HIV-1 PAP with Saquinavir (Figure 7E,F). To provide a more intuitive analysis of binding modes, we drew a ligand interaction diagram. As shown in Figure 7F, the hydroxyl groups of saquinavir all form hydrogen bonding interactions with the catalytic residue Asp of proteases, which is essential to the binding of PAP inhibitors to the protein. Inhibitors can also form salt bridges with the catalytic residue Asp of proteases. Inhibitors also have various interactions with other parts of proteases, such as hydrophobic interaction, *π*-*π* interaction, and hydrogen bond interaction. Overall, HIV protease inhibitors have the potential to inhibit the other nine retroviruses.

## 3. Discussion and Conclusions

In this study, we collected and clustered 170 protease sequences from 125 viruses related to the infection of humans. The sequences, structures, and substrate characteristics of the viral 3CL^pro^ and PAPs were analyzed. We employed a ligand-protein complex structure prediction method to reposition approved drugs for viral 3CL^pro^ and PAPs. Taking Ray1216 and Asunaprevir as examples, we modified and designed inhibitors for the DENV protease and conducted computational simulations. We classified proteases from various viruses, offering valuable insights for systematic research and the design of antiviral drugs.

There is no specific drug available to treat viruses in the order Picornavirales. For example, HRV is a virus in the order Picornavirales. The clinical trial of the HRV inhibitor (Rupintrivir) was terminated due to low oral bioavailability [45]. Our results indicate that four approved drugs targeting the 3CL^pro^ of SARS-CoV-2 show potential for inhibiting the proteases of multiple viruses in the order Picornavirales (Figure 1 and Figure 4E). Expanding the indications of these drugs can help address the current shortage of specific medications for Picornavirales.

In addition, Ensitrelvir is a non-peptidomimetic, non-covalent inhibitor that targets the SARS-CoV-2 3CL^pro^. As depicted in Figure S8, the predicted complex structure of DENV NS3/2B-Ensitrelvir is quite similar to the complex structure of SARS-CoV-2 3CL^pro^-Ensitrelvir. The P1’, P1, and P2 segments of Ensitrelvir are well bound with the S1’, S1, and S2 sites in both complex structures. Ensitrelvir is a potential inhibitor targeting DENV NS3/2B.

Furthermore, our study has demonstrated that the strategy is suitable for homologous proteins with significant differences. The RMSD between SARS-CoV-2 3CL^pro^ with SIM0417 (PDB code: 8IGX) and MODV NS3/2B is 6.226 Å. The RMSD between HCV NS3/4A with Boceprevir (PDB code: 3LOX) and PeV 3CL^pro^ is 7.539 Å. The RMSD between HIV-1 Protease with Saquinavir (PDB code: 4QGI) and SFV Protease is 4.739 Å. The RMSD values between proteins are significant. As shown in Figure S9, we displayed and compared the complex structures. There are many unreasonable interactions between small molecules extracted from the experimentally determined structures and the target proteins (Figure S9B). After refining the complex structures in Figure S9B using our complex structure prediction strategy, we obtained the predicted complex structures of MODV NS3/2B-SIM0417, PeV 3CL^pro^-Boceprevir, and SFV Protease-Saquinavir (Figure S9C). The P4’-P4 segments of ligands occupy well with the S4-S4’ sites on the surface of proteins.

This study proposed the potential of repurposing anti-virus drugs for other known viruses that contain similar proteins and provided modification strategies for further optimizing these drugs. For example, inhibitors of the VEEV nsP2 protease can be used to find inhibitors for other alphavirus nsP2 proteins [78]. The inhibitors of HCMV and HHV6 assemblins can be applied to finding inhibitors of other Herpesvirus assemblins [79]. The folding of Poxviridae core proteases is similar to that of Adenoviridae adenains. The inhibitors of Adenoviridae adenains have been reported [80,81,82]. Using this as a reference, inhibitors of Poxviridae core proteases can be designed. However, our strategy also has some limitations. Compared to molecular dynamics simulations with larger sampling [83], this strategy is faster but cannot provide as much useful information. This limitation needs to be considered in the future. Typically, active sites are highly conservative, whereas allosteric sites are relatively unique, so our strategy may not be as applicable to allosteric sites. In addition, we only selected one sequence for each virus. In fact, each virus exists in multiple mutated forms. Research on virus resistance is also especially important.

With the increasing availability of information about viruses, there is a growing need for systematic research on viruses and improved treatment of antiviral infections. Exploiting broad-spectrum inhibitors for similar viral targets and summarizing inhibitor design strategies for the same type of target will advance the development of antiviral drugs.

## 4. Materials and Methods

### 4.1. Data Collection

It is reported that 263 viruses are known to infect humans [4,5]. We introduced seven other viruses, including SARS-CoV-2, HCoV-HKU1, HCoV-NL63, CCoV-HuPn-2018, Hu-PDCoV, and HTLV-4 [6,7,8,9,10]. There are around 125 viruses that can express proteases. We collected a total of 170 viral protease sequences. The protease sequences were retrieved from ViralZone [84], the NCBI database (https://www.ncbi.nlm.nih.gov/, accessed on 1 August 2023), the Protein Data Bank (PDB) [85], and UniProt [86]. The substrate sequences originated from the NCBI database. The seq-logo of substrates was depicted by WebLogo [87]. The abbreviation and full name are shown in Abbreviations. The access IDs of these viruses are added to the Supplementary Materials.

The PDB codes of 3CL^pro^ with Nirmatrelvir, Ensitrelvir, SIM0417, RAY1216, Boceprevir, Telaprevir, Narlaprevir, Asunaprevir, Danoprevir, Simeprevir, Vaniprevir, Grazoprevir, Voxilaprevir, and Glecaprevir were 7RFS, 7VU6, 8IGX, 8IGN, 3LOX, 3SV6, 3LON, 4WF8, 5EQR, 3KEE, 3SU3, 6P6R, 6NZT, and 3SUD, respectively [16,18,19,20,30,88,89,90,91,92]. The PDB codes of PAPs with Saquinavir, Indinavir, Ritonavir, Nelfinavir, Amprenavir, Lopinavir, Atazanavir, Tipranavir, and Darunavir were 4QGI, 2B7Z, 4EYR, 2R5Q, 3S45, 1MUI, 2FXD, 6DIF, and 4LL3, respectively [93,94,95,96,97,98,99,100].

### 4.2. The Protease Profiles of Human-Infective Viruses

The clan classification was based on the MEROPS database [101]. The proteases in the same clan were clustered by phylogenetic analysis in MEGA 11 [102]. The sequences were first aligned by ClustalW [103]. The model selection was analyzed in the Find Best DNA/Protein Models (ML) option in MEGA 11. The model with the lowest BIC for model parameters was selected. The best-fit models of clan PA, clan CA, and clan SH are WAG + G models. The best-fit model of clan AA is the rtREV + G + I model. The best-fit models of clan CN, clan CE, and clan ME are LG + G models. The phylogenetic analysis was conducted using the Maximum Likelihood method. The dendrogram was generated by Evolview V3 [104].

### 4.3. Sequence Alignment

The identity matrixes of aligned sequences by ClustalW were calculated by the BioAider tool [105]. The heat map was drawn with GraphPad Prism 8.0 software. The structure-based multiple sequence alignment was conducted by PROMALS3D [106]. The seq-logo of aligned sequences was depicted by WebLogo [87].

### 4.4. Three-Dimensional Structure Prediction

Protein structures and complexes were predicted by ColabFold [71]. Choose the template structures with the highest rank. The structures of Flaviviridae NS3/4A, NS3/2B, and Retrovirales proteases are dimers. The structures of other sequences are monomeric. Due to the predicted structures of HEV PL^pro^, BToV 3CL^pro^, and BToV PL^pro^ with low confidence, the proteins were removed from the proteases list. The PDB ID of proteins with reported structures is added to the Supplementary Materials.

### 4.5. The Computational Ligand-Protein Complex Structure Prediction

The protein structures were first prepared. Solvents and ligands were removed from the experimentally determined structures. One chain of proteins with multiple chains retained. We use the Protein Preparation Wizard in Maestro 12.8 (Schrödinger, LLC, New York, NY, USA, 2021) to assign correct protonation states and formal charges and add missing residues [107].

The pipeline for complex structure prediction is shown in Figure 4D. For proteins without experimental complex structures, the structures were constructed following the steps of aligning the protein structures, extracting ligands into target protein structures, and performing local refinement for complex structures. For ligands without experimental complex structures, the steps will be modified as follows: aligning the target ligands into the reference ligands, extracting target proteins, merging proteins and target ligands, and performing local refinement for complex structures.

For example, the complex structure of HCV 3CL^pro^ with Paritaprevir has not been experimentally determined, but the 2D structure of Paritaprevir is similar to that of Danoprevir. Paritaprevir was preprocessed by using the LigPrep module in Maestro 12.8 to predict the protonation state and generate the low-energetical 3D conformation. The structure of Paritaprevir was aligned to the ligand Danoprevir in PDB 5EQR by Ligand Alignment in Maestro 12.8. Then, the complex structure of HCV 3CL^pro^ with Paritaprevir was constructed by Prime MM-GBSA in Maestro 12.8. The compounds **1**–**2**, designed by us, were simulated using the same methods.

The 3CL^pro^ or PAPs of multiple viruses were aligned to the above complex structures by Cealign in PyMOL [68,69]. Then, the complex structures of proteins with ligands were constructed, and the binding energies were calculated by Prime MM-GBSA. The flexible residue distance from the ligand is 5 Å. The solvation model VSGB and force field OPLS4 were used in the simulations. Heat maps of energy matrixes were drawn with GraphPad Prism 8.0 software.

## Figures and Tables

**Table 1:** The grouped clans, catalytic residues, and the description of the function in the viral life cycle of viral proteases.

Clans	Description	Proteases	Catalytic Residues	Functions
Clan PA	Proteases of mixed nucleophiles, superfamily A (Cysteine or Serine proteases)	3CL^pro^	His-Cys His-Asn/Glu-Cys His-Asn/Glu-Ser	Cleave the polyprotein Structural protein
Clan CA	Cysteine peptidase, superfamily A	PL^pro^	Cys-His-Asn/Asp	Cleave the polyprotein Structural protein DUBs activity
Clan CE	Cysteine peptidase, superfamily E	Poxviridae core protease Adenoviridae adenain	His-Glu/Asp-Cys	Structural protein Viral assembly
Clan CN	Cysteine peptidase, superfamily N	Alphavirus nsP2	Cys-His	Cleave the polyprotein
Clan SH	Serine peptidase, superfamily H	Herpesvirus assemblin	His-Ser-His	Structural protein Viral assembly
Clan AA	Aspartic peptidase, superfamily A	PAPs	Asp-Asp	Cleave the polyprotein Structural protein
Clan ME	Metallo-peptidase, superfamily E	Poxviridae Metallo-protease	His-Xaa-Xaa-Glu-His	Virion maturation

## Data Availability

Data are contained within this article.

## Supplementary Materials

The following supporting information can be downloaded at: https://www.mdpi.com/article/10.3390/molecules29010225/s1, Figure S1: The PROMALS3D alignment of viral 3CL^pro^; Figure S2: The substrate sequences of 13 classes of viruses 3CL^pro^; Figure S3: The active sites comparison of viral 3CL^pro^; Figure S4: The predicted binding modes of SARS-CoV-2 3CL^pro^ with Vaniprevir or Simeprevir; Figure S5: Predicted binding modes of HAtV, DENV, HCV, and CHIKV 3CL^pro^ with Ray1216 [58,108]; Figure S6: The PROMALS3D alignment of viral PAPs; Figure S7: The sequences, substrates, and RMSD values of viral PAPs; Figure S8: The complex structures of SARS-CoV-2 3CL^pro^-Ensitrelvir and DENV NS3/2B Ensitrelvir [20,71]; Figure S9: The complex structures of viral proteases with protease inhibitors [18,71,88,93]; Table S1: The approved small-molecule drugs targeting eleven viruses Excel: The access ID, RMSD, and dG of viral proteases.

## Abbreviations

AiV	Aichi virus	HuNoV	Human norovirus
AROAV	Aroa virus	Hu-PDCoV	Human-Porcine Delta coronavirus
AURAV	Aura virus	ILHV	Ilheus virus
BANV	Banzi virus	JEV	Japanese encephalitis virus
BEBV	Bebaru virus	KFDV	Kyasanur Forest disease virus
BFV	Barmah Forest virus	KOKV	Kokobera virus
BLV	Bovine leukemia virus	KOUV	Koutango virus
BOUV	Bouboui virus	LIV	Louping ill virus
BPSV	Bovine papular stomatitis virus	LjV	Ljungan virus
BToV	Bovine torovirus	MAYV	Mayaro virus
BV	Cercopithecine herpesvirus 1	MCV-1	Molluscum contagiosum virus
CCHFV	Crimean-Congo hemorrhagic fever virus	MDPV	Mosso das Pedras virus
CCoV-HuPn-2018	Canine coronavirus-human pneumonia-2018	MERS-CoV	Middle East respiratory syndrome
CHIKV	Chikungunya virus	MODV	Modoc virus
CMLV	Camelpox virus	MPXV	Monkeypox virus
CPCV	Cacipacore virus	MVEV	Murray Valley encephalitis virus
CPXV	Cowpox virus	NDUV	Ndumu virus
DENV	Dengue virus	NTAV	Ntaya virus
EBV	Human herpesvirus 4 (Epstein-Barr Virus)	NV	Norwalk virus
EEEV	Eastern equine encephalitis virus	OHFV	Omsk hemorrhagic fever virus
EMCV	Encephalomyocarditis virus	ONNV	O’nyong-nyong virus
EV-A71	Enterovirus A71	ORFV	Orf virus
EV-B93	Enterovirus B93	PCPV	Pseudocowpox virus
EV-C	Enterovirus C	PeV	Parechovirus
EV-D68	Enterovirus D68	PIXV	Pixuna virus
EVEV	Everglades virus	POWV	Powassan virus
FMDV-C	Foot-and-mouth disease virus C	PV	Poliovirus (EV-C)
GaLV	Gibbon ape leukemia virus	RBV	Rio Bravo virus
GBV C	GB virus C	RNV	Rio Negro virus
GETV	Getah virus	RRV	Ross River virus
HAdV-A	Human adenovirus A	RV	Rubella virus
HAdV-B	Human adenovirus B	SARS-CoV	Severe acute respiratory syndrome
HAdV-C	Human adenovirus C	SARS-CoV-2	Severe acute respiratory syndrome coronavirus 2
HAdV-D	Human adenovirus D	SaV	Sapporo virus
HAdV-E	Human adenovirus E	SFV	Simian foamy virus
HAdV-F	Human adenovirus F	SFV	Semliki Forest virus
HAtV	Human astrovirus	SINV	Sindbis virus
HAV	Hepatitis A virus	SLEV	St. Louis encephalitis virus
HCoV 229E	Human coronavirus 229E	SPPV	Sealpox virus
HCoV HKU1	Human coronavirus HKU1	SuHV-1	Suid herpesvirus 1
HCoV NL63	Human coronavirus NL63	TBEV	Tick-borne encephalitis virus
HCoV OC43	Human coronavirus OC43	TMUV	Tembusu virus
HCV	Hepatitis C Virus	TONV	Tonate virus
HHV-5/HCMV	Human herpesvirus 5	TPV	Tanapox virus
HHV-6	Human herpesvirus 6	UNVA	Una virus
HHV-7	Human herpesvirus 7	USUV	Usutu virus
HHV-8	Human herpesvirus 8	VACV	Vaccinia virus
HIV-1	Human immunodeficiency virus 1	VARV	Variola virus
HIV-2	Human immunodeficiency virus 2	VEEV	Venezuelan equine encephalitis virus
HRV-A	Human rhinovirus A	WEEV	Western equine encephalitis virus
HRV-B	Human rhinovirus B	WMSV	Woolly monkey sarcoma virus
HSV-1	Human herpesvirus 1	WNV	West Nile virus
HSV-2	Human herpesvirus 2	WSLV	Wesselsbron virus
HSV-3 (VZV)	Human herpesvirus 3 (Varicella-zostervirus)	XMRV	Xenotropic murine leukemia virus-related virus
HTLV-1	Primate T-lymphotropic virus 1	YFV	Yellow fever virus
HTLV-2	Primate T-lymphotropic virus 2	YMTV	Yaba monkey tumor virus
HTLV-3	Primate T-lymphotropic virus 3	ZIKV	Zika virus
HTLV-4	Primate T-lymphotropic virus 4		

## References

[1] Zhu N., Zhang D., Wang W., Li X., Yang B., Song J., Zhao X., Huang B., Shi W., Lu R. (2020). A novel coronavirus from patients with pneumonia in China, 2019. N. Engl. J. Med..

[2] Wang C., Horby P.W., Hayden F.G., Gao G.F. (2020). A novel coronavirus outbreak of global health concern. Lancet.

[3] Statement on the Fifteenth Meeting of the IHR (2005) Emergency Committee on the COVID-19 Pandemic. https://www.who.int/news/item/05-05-2023-statement-on-the-fifteenth-meeting-of-the-international-health-regulations-(2005)-emergency-committee-regarding-the-coronavirus-disease-(covid-19)-pandemic.

[4] Carroll D., Daszak P., Wolfe N.D., Gao G.F., Morel C.M., Morzaria S., Pablos-Méndez A., Tomori O., Mazet J.A.K. (2018). The Global Virome Project. Science.

[5] Olival K.J., Hosseini P.R., Zambrana-Torrelio C., Ross N., Bogich T.L., Daszak P. (2017). Host and viral traits predict zoonotic spillover from mammals. Nature.

[6] Mahieux R., Gessain A. (2009). The human HTLV-3 and HTLV-4 retroviruses: New members of the HTLV family. Pathol. Biol..

[7] Lednicky J.A., Tagliamonte M.S., White S.K., Elbadry M.A., Alam M.M., Stephenson C.J., Bonny T.S., Loeb J.C., Telisma T., Chavannes S. (2021). Independent infections of porcine deltacoronavirus among Haitian children. Nature.

[8] Van der Hoek L., Pyrc K., Jebbink M.F., Vermeulen-Oost W., Berkhout R.J.M., Wolthers K.C., Wertheim-van Dillen P.M.E., Kaandorp J., Spaargaren J., Berkhout B. (2004). Identification of a new human coronavirus. Nat. Med..

[9] Vlasova A.N., Diaz A., Damtie D., Xiu L., Toh T.-H., Lee J.S.-Y., Saif L.J., Gray G.C. (2021). Novel canine coronavirus isolated from a hospitalized patient with pneumonia in east malaysia. Clin. Infect. Dis..

[10] Woo P.C.Y., Lau S.K.P., Yip C.C.Y., Huang Y., Yuen K.-Y. (2009). More and more coronaviruses: Human coronavirus HKU1. Viruses.

[11] Tompa D.R., Immanuel A., Srikanth S., Kadhirvel S. (2021). Trends and strategies to combat viral infections: A review on FDA approved antiviral drugs. Int. J. Biol. Macromol..

[12] Jin Z., Du X., Xu Y., Deng Y., Liu M., Zhao Y., Zhang B., Li X., Zhang L., Peng C. (2020). Structure of Mpro from SARS-CoV-2 and discovery of its inhibitors. Nature.

[13] Hijikata M., Mizushima H., Tanji Y., Komoda Y., Hirowatari Y., Akagi T., Kato N., Kimura K., Shimotohno K. (1993). Proteolytic processing and membrane association of putative nonstructural proteins of hepatitis C virus. Proc. Natl. Acad. Sci. USA.

[14] Raney K.D., Sharma S.D., Moustafa I.M., Cameron C.E. (2010). Hepatitis C virus non-structural protein 3 (HCV NS3): A multifunctional antiviral target. J. Biol. Chem..

[15] Brik A., Wong C.-H. (2003). HIV-1 protease: Mechanism and drug discovery. Org. Biomol. Chem..

[16] Owen D.R., Allerton C.M.N., Anderson A.S., Aschenbrenner L., Avery M., Berritt S., Boras B., Cardin R.D., Carlo A., Coffman K.J. (2021). An oral SARS-CoV-2 Mpro inhibitor clinical candidate for the treatment of COVID-19. Science.

[17] Hammond J., Leister-Tebbe H., Gardner A., Abreu P., Bao W., Wisemandle W., Baniecki M., Hendrick V.M., Damle B., Simón-Campos A. (2022). Oral Nirmatrelvir for high-risk, nonhospitalized adults with COVID-19. N. Engl. J. Med..

[18] Jiang X., Su H., Shang W., Zhou F., Zhang Y., Zhao W., Zhang Q., Xie H., Jiang L., Nie T. (2023). Structure-based development and preclinical evaluation of the SARS-CoV-2 3C-like protease inhibitor simnotrelvir. Nat. Commun..

[19] Chen X., Huang X., Ma Q., Kuzmič P., Zhou B., Xu J., Liu B., Jiang H., Zhang W., Yang C. (2023). Inhibition mechanism and antiviral activity of an α-ketoamide based SARS-CoV-2 main protease inhibitor. bioRxiv.

[20] Unoh Y., Uehara S., Nakahara K., Nobori H., Yamatsu Y., Yamamoto S., Maruyama Y., Taoda Y., Kasamatsu K., Suto T. (2022). Discovery of S-217622, a noncovalent oral SARS-CoV-2 3CL protease inhibitor clinical candidate for treating COVID-19. J. Med. Chem..

[21] Bacon B.R., Gordon S.C., Lawitz E., Marcellin P., Vierling J.M., Zeuzem S., Poordad F., Goodman Z.D., Sings H.L., Boparai N. (2011). Boceprevir for previously treated chronic HCV genotype 1 infection. N. Engl. J. Med..

[22] Lin C., Kwong D.A., Perni B.R. (2006). Discovery and development of VX-950, a novel, covalent, and reversible inhibitor of Hepatitis C virus NS3.4A serine protease. Infect. Disord.—Drug Targets.

[23] Arasappan A., Bennett F., Bogen S.L., Venkatraman S., Blackman M., Chen K.X., Hendrata S., Huang Y., Huelgas R.M., Nair L. (2010). Discovery of narlaprevir (SCH 900518): A potent, second generation HCV NS3 serine protease inhibitor. ACS Med. Chem. Lett..

[24] Lok A.S., Gardiner D.F., Lawitz E., Martorell C., Everson G.T., Ghalib R., Reindollar R., Rustgi V., McPhee F., Wind-Rotolo M. (2012). Preliminary study of two antiviral agents for Hepatitis C genotype 1. N. Engl. J. Med..

[25] Jiang Y., Andrews S.W., Condroski K.R., Buckman B., Serebryany V., Wenglowsky S., Kennedy A.L., Madduru M.R., Wang B., Lyon M. (2014). Discovery of Danoprevir (ITMN-191/R7227), a highly selective and potent inhibitor of Hepatitis C Virus (HCV) NS3/4A protease. J. Med. Chem..

[26] Gentile I., Borgia F., Buonomo R.A., Zappulo E., Castaldo G., Borgia G. (2014). ABT-450: A novel protease inhibitor for the treatment of Hepatitis C Virus infection. Curr. Med. Chem..

[27] Lin T.-I., Lenz O., Fanning G., Verbinnen T., Delouvroy F., Scholliers A., Vermeiren K., Rosenquist Å., Edlund M., Samuelsson B. (2009). In Vitro activity and preclinical profile of TMC435350, a potent Hepatitis C Virus protease inhibitor. Antimicrob. Agents Chemother..

[28] McCauley J.A., McIntyre C.J., Rudd M.T., Nguyen K.T., Romano J.J., Butcher J.W., Gilbert K.F., Bush K.J., Holloway M.K., Swestock J. (2010). Discovery of Vaniprevir (MK-7009), a macrocyclic Hepatitis C Virus NS3/4a protease inhibitor. J. Med. Chem..

[29] Harper S., McCauley J.A., Rudd M.T., Ferrara M., DiFilippo M., Crescenzi B., Koch U., Petrocchi A., Holloway M.K., Butcher J.W. (2012). Discovery of MK-5172, a macrocyclic Hepatitis C Virus NS3/4a protease inhibitor. ACS Med. Chem. Lett..

[30] Taylor J.G., Zipfel S., Ramey K., Vivian R., Schrier A., Karki K.K., Katana A., Kato D., Kobayashi T., Martinez R. (2019). Discovery of the pan-genotypic hepatitis C virus NS3/4A protease inhibitor voxilaprevir (GS-9857): A component of Vosevi^®^. Bioorganic Med. Chem. Lett..

[31] Ng Teresa I., Tripathi R., Reisch T., Lu L., Middleton T., Hopkins Todd A., Pithawalla R., Irvin M., Dekhtyar T., Krishnan P. (2017). In Vitro antiviral activity and resistance profile of the next-generation Hepatitis C Virus NS3/4A protease inhibitor Glecaprevir. Antimicrob. Agents Chemother..

[32] Zephyr J., Nageswara Rao D., Vo S.V., Henes M., Kosovrasti K., Matthew A.N., Hedger A.K., Timm J., Chan E.T., Ali A. (2022). Deciphering the molecular mechanism of HCV protease inhibitor Fluorination as a general approach to avoid drug resistance. J. Mol. Biol..

[33] Gallo R.C., Sarin P.S., Gelmann E.P., Robert-Guroff M., Richardson E., Kalyanaraman V.S., Mann D., Sidhu G.D., Stahl R.E., Zolla-Pazner S. (1983). Isolation of Human T-Cell Leukemia Virus in Acquired Immune Deficiency Syndrome (AIDS). Science.

[34] James J.S. (1995). Saquinavir (Invirase): First protease inhibitor approved--reimbursement, information hotline numbers. AIDS Treat. News.

[35] Kempf D.J., Sham H.L., Marsh K.C., Flentge C.A., Betebenner D., Green B.E., McDonald E., Vasavanonda S., Saldivar A., Wideburg N.E. (1998). Discovery of Ritonavir, a potent inhibitor of HIV protease with high oral bioavailability and clinical efficacy. J. Med. Chem..

[36] Vacca J.P., Dorsey B.D., Schleif W.A., Levin R.B., McDaniel S.L., Darke P.L., Zugay J., Quintero J.C., Blahy O.M., Roth E. (1994). L-735,524: An orally bioavailable human immunodeficiency virus type 1 protease inhibitor. Proc. Natl. Acad. Sci. USA.

[37] Kaldor S.W., Kalish V.J., Davies J.F., Shetty B.V., Fritz J.E., Appelt K., Burgess J.A., Campanale K.M., Chirgadze N.Y., Clawson D.K. (1997). Viracept (Nelfinavir Mesylate, AG1343):  a potent, orally bioavailable inhibitor of HIV-1 protease. J. Med. Chem..

[38] Clair M.S., Millard J., Rooney J., Tisdale M., Parry N., Sadler B.M., Blum M.R., Painter G. (1996). In vitro antiviral activity of 141W94 (VX-478) in combination with other antiretroviral agents. Antivir. Res..

[39] Sham H.L., Kempf D.J., Molla A., Marsh K.C., Kumar G.N., Chen C.-M., Kati W., Stewart K., Lal R., Hsu A. (1998). ABT-378, a highly potent inhibitor of the Human Immunodeficiency Virus protease. Antimicrob. Agents Chemother..

[40] Robinson B.S., Riccardi K.A., Gong Y.-f., Guo Q., Stock D.A., Blair W.S., Terry B.J., Deminie C.A., Djang F., Colonno R.J. (2000). BMS-232632, a highly potent Human Immunodeficiency Virus protease inhibitor that can be used in combination with other available antiretroviral agents. Antimicrob. Agents Chemother..

[41] Flexner C., Bate G., Kirkpatrick P. (2005). Tipranavir. Nat. Rev. Drug Discov..

[42] Molina J.-M., Hill A. (2007). Darunavir (TMC114): A new HIV-1 protease inhibitor. Expert Opin. Pharmacother..

[43] Gao Y., Sun S.-Q., Guo H.-C. (2016). Biological function of Foot-and-mouth disease virus non-structural proteins and non-coding elements. Virol. J..

[44] Wang J., Hu Y., Zheng M. (2022). Enterovirus A71 antivirals: Past, present, and future. Acta Pharm. Sin. B.

[45] Patick A.K., Binford S.L., Brothers M.A., Jackson R.L., Ford C.E., Diem M.D., Maldonado F., Dragovich P.S., Zhou R., Prins T.J. (1999). In Vitro antiviral activity of AG7088, a potent inhibitor of Human Rhinovirus 3C protease. Antimicrob. Agents Chemother..

[46] Kim Y., Galasiti Kankanamalage A.C., Chang K.-O., Groutas W.C. (2015). Recent advances in the discovery of Norovirus therapeutics. J. Med. Chem..

[47] Behnam M.A.M., Nitsche C., Boldescu V., Klein C.D. (2016). The medicinal chemistry of Dengue Virus. J. Med. Chem..

[48] Lim Y.X., Ng Y.L., Tam J.P., Liu D.X. (2016). Human Coronaviruses: A review of virus–host interactions. Diseases.

[49] Melancon P., Garoff H. (1987). Processing of the Semliki Forest virus structural polyprotein: Role of the capsid protease. J. Virol..

[50] Skoging U., Liljeström P. (1998). Role of the C-terminal tryptophan residue for the structure-function of the alphavirus capsid protein11Edited by J. Karn. J. Mol. Biol..

[51] Thomas S., Rai J., John L., Günther S., Drosten C., Pützer B.M., Schaefer S. (2010). Functional dissection of the alphavirus capsid protease: Sequence requirements for activity. Virol. J..

[52] Yi J., Peng J., Yang W., Zhu G., Ren J., Li D., Zheng H. (2021). Picornavirus 3C—A protease ensuring virus replication and subverting host responses. J. Cell Sci..

[53] Speroni S., Rohayem J., Nenci S., Bonivento D., Robel I., Barthel J., Luzhkov V.B., Coutard B., Canard B., Mattevi A. (2009). Structural and biochemical analysis of human pathogenic Astrovirus serine protease at 2.0 Å resolution. J. Mol. Biol..

[54] Shin G., Yost S.A., Miller M.T., Elrod E.J., Grakoui A., Marcotrigiano J. (2012). Structural and functional insights into alphavirus polyprotein processing and pathogenesis. Proc. Natl. Acad. Sci. USA.

[55] Yuan S., Gao X., Tang K., Cai J.-P., Hu M., Luo P., Wen L., Ye Z.-W., Luo C., Tsang J.O.-L. (2022). Targeting papain-like protease for broad-spectrum coronavirus inhibition. Protein Cell.

[56] Bailey-Elkin B.A., Knaap R.C.M., Kikkert M., Mark B.L. (2017). Structure and function of viral deubiquitinating enzymes. J. Mol. Biol..

[57] Mielech A.M., Chen Y., Mesecar A.D., Baker S.C. (2014). Nidovirus papain-like proteases: Multifunctional enzymes with protease, deubiquitinating and deISGylating activities. Virus Res..

[58] Durie I.A., Dzimianski J.V., Daczkowski C.M., McGuire J., Faaberg K., Pegan S.D. (2021). Structural insights into the interaction of papain-like protease 2 from the alphacoronavirus porcine epidemic diarrhea virus and ubiquitin. Acta Crystallogr. D Struct. Biol..

[59] Cheong E.Z.K., Quek J.P., Xin L., Li C., Chan J.Y., Liew C.W., Mu Y., Zheng J., Luo D. (2022). Crystal structure of the Rubella virus protease reveals a unique papain-like protease fold. J. Biol. Chem..

[60] Tchesnokov E.P., Bailey-Elkin B.A., Mark B.L., Götte M. (2020). Independent inhibition of the polymerase and deubiquitinase activities of the Crimean-Congo Hemorrhagic Fever Virus full-length L-protein. PLoS Neglected Trop. Dis..

[61] Zühlsdorf M., Hinrichs W. (2017). Assemblins as maturational proteases in herpesviruses. J. Gen. Virol..

[62] Hall D.L., Darke P.L. (1995). Activation of the Herpes Simplex Virus Type 1 Protease (∗). J. Biol. Chem..

[63] Harrison J.J.E.K., Tuske S., Das K., Ruiz F.X., Bauman J.D., Boyer P.L., DeStefano J.J., Hughes S.H., Arnold E. (2021). Crystal structure of a Retroviral polyprotein: Prototype Foamy Virus Protease-Reverse Transcriptase (PR-RT). Viruses.

[64] Chen J.-S., Li H.-C., Lin S.-I., Yang C.-H., Chien W.-Y., Syu C.-L., Lo S.-Y. (2015). Cleavage of dicer protein by I7 protease during vaccinia virus infection. PLoS ONE.

[65] Ansarah-Sobrinho C., Moss B. (2004). Role of the I7 protein in proteolytic processing of vaccinia virus membrane and core components. J. Virol..

[66] Ruzindana-Umunyana A., Sircar S., Weber J.M. (2000). The effect of mutant peptide cofactors on Adenovirus protease activity and virus infection. Virology.

[67] Hedengren-Olcott M., Byrd C.M., Watson J., Hruby D.E. (2004). The Vaccinia Virus G1L putative Metalloproteinase is essential for viral replication in vivo. J. Virol..

[68] DeLano W.L. (2002). Pymol: An open-source molecular graphics tool. CCP4 Newsl. Protein Crystallogr..

[69] Shindyalov I.N., Bourne P.E. (1998). Protein structure alignment by incremental combinatorial extension (CE) of the optimal path. Protein Eng..

[70] Jumper J., Evans R., Pritzel A., Green T., Figurnov M., Ronneberger O., Tunyasuvunakool K., Bates R., Žídek A., Potapenko A. (2021). Highly accurate protein structure prediction with AlphaFold. Nature.

[71] Mirdita M., Schütze K., Moriwaki Y., Heo L., Ovchinnikov S., Steinegger M. (2022). ColabFold: Making protein folding accessible to all. Nat. Methods.

[72] Joseph M.H., Georgios A., Lichang W. (2012). MM-GB(PB)SA calculations of protein-ligand binding free energies. Molecular Dynamics.

[73] Pushpakom S., Iorio F., Eyers P.A., Escott K.J., Hopper S., Wells A., Doig A., Guilliams T., Latimer J., McNamee C. (2019). Drug repurposing: Progress, challenges and recommendations. Nat. Rev. Drug Discov..

[74] Zunszain P.A., Knox S.R., Sweeney T.R., Yang J., Roqué-Rosell N., Belsham G.J., Leatherbarrow R.J., Curry S. (2010). Insights into cleavage specificity from the crystal structure of Foot-and-Mouth Disease Virus 3C protease complexed with a peptide substrate. J. Mol. Biol..

[75] Lu G., Qi J., Chen Z., Xu X., Gao F., Lin D., Qian W., Liu H., Jiang H., Yan J. (2011). Enterovirus 71 and Coxsackievirus A16 3C proteases: Binding to Rupintrivir and their substrates and anti-Hand, Foot, and Mouth Disease Virus drug design. J. Virol..

[76] Yin J., Cherney M.M., Bergmann E.M., Zhang J., Huitema C., Pettersson H., Eltis L.D., Vederas J.C., James M.N.G. (2006). An episulfide cation (thiiranium ring) trapped in the active site of HAV 3C proteinase inactivated by peptide-based Ketone inhibitors. J. Mol. Biol..

[77] Galasiti Kankanamalage A.C., Kim Y., Rathnayake A.D., Damalanka V.C., Weerawarna P.M., Doyle S.T., Alsoudi A.F., Dissanayake D.M.P., Lushington G.H., Mehzabeen N. (2017). Structure-based exploration and exploitation of the S4 subsite of norovirus 3CL protease in the design of potent and permeable inhibitors. Eur. J. Med. Chem..

[78] Hu X., Morazzani E., Compton J.R., Harmon M., Soloveva V., Glass P.J., Garcia A.D., Marugan J.J., Legler P.M. (2023). In silico screening of inhibitors of the Venezuelan Equine Encephalitis Virus nonstructural protein 2 cysteine protease. Viruses.

[79] Yoshida S., Sako Y., Nikaido E., Ueda T., Kozono I., Ichihashi Y., Nakahashi A., Onishi M., Yamatsu Y., Kato T. (2023). Peptide-to-small molecule: Discovery of non-covalent, active-site inhibitors of β-Herpesvirus proteases. ACS Med. Chem. Lett..

[80] Grosche P., Sirockin F., Mac Sweeney A., Ramage P., Erbel P., Melkko S., Bernardi A., Hughes N., Ellis D., Combrink K.D. (2015). Structure-based design and optimization of potent inhibitors of the adenoviral protease. Bioorganic Med. Chem. Lett..

[81] Mac Sweeney A., Grosche P., Ellis D., Combrink K., Erbel P., Hughes N., Sirockin F., Melkko S., Bernardi A., Ramage P. (2014). Discovery and structure-based optimization of Adenain inhibitors. ACS Med. Chem. Lett..

[82] McGrath W.J., Ding J., Didwania A., Sweet R.M., Mangel W.F. (2003). Crystallographic structure at 1.6-Å resolution of the human adenovirus proteinase in a covalent complex with its 11-amino-acid peptide cofactor: Insights on a new fold. Biochim. Et Biophys. Acta (BBA)—Proteins Proteom..

[83] Sinha S., Tam B., Wang S.M. (2022). Applications of molecular dynamics simulation in protein study. Membranes.

[84] Hulo C., de Castro E., Masson P., Bougueleret L., Bairoch A., Xenarios I., Le Mercier P. (2011). ViralZone: A knowledge resource to understand virus diversity. Nucleic Acids Res..

[85] Berman H.M., Westbrook J., Feng Z., Gilliland G., Bhat T.N., Weissig H., Shindyalov I.N., Bourne P.E. (2000). The Protein Data Bank. Nucleic Acids Res..

[86] (2023). UniProt: The Universal Protein Knowledgebase in 2023. Nucleic Acids Res.

[87] Crooks G.E., Hon G., Chandonia J.M., Brenner S.E. (2004). WebLogo: A sequence logo generator. Genome Res..

[88] Bennett F., Huang Y., Hendrata S., Lovey R., Bogen S.L., Pan W., Guo Z., Prongay A., Chen K.X., Arasappan A. (2010). The introduction of P4 substituted 1-methylcyclohexyl groups into Boceprevir®: A change in direction in the search for a second generation HCV NS3 protease inhibitor. Bioorganic Med. Chem. Lett..

[89] Romano K.P., Ali A., Aydin C., Soumana D., Özen A., Deveau L.M., Silver C., Cao H., Newton A., Petropoulos C.J. (2012). The molecular basis of drug resistance against Hepatitis C Virus NS3/4A protease inhibitors. PLoS Pathog..

[90] Soumana D.I., Ali A., Schiffer C.A. (2014). Structural analysis of Asunaprevir resistance in HCV NS3/4A protease. ACS Chem. Biol..

[91] Soumana D.I., Kurt Yilmaz N., Ali A., Prachanronarong K.L., Schiffer C.A. (2016). Molecular and dynamic mechanism underlying drug resistance in genotype 3 Hepatitis C NS3/4A protease. J. Am. Chem. Soc..

[92] Cummings M.D., Lindberg J., Lin T.-I., de Kock H., Lenz O., Lilja E., Felländer S., Baraznenok V., Nyström S., Nilsson M. (2010). Induced-fit binding of the macrocyclic noncovalent inhibitor TMC435 to its HCV NS3/NS4A protease target. Angew. Chem. Int. Ed..

[93] Goldfarb N.E., Ohanessian M., Biswas S., McGee T.D., Mahon B.P., Ostrov D.A., Garcia J., Tang Y., McKenna R., Roitberg A. (2015). Defective hydrophobic sliding mechanism and active site expansion in HIV-1 protease drug resistant variant Gly48Thr/Leu89Met: Mechanisms for the loss of Saquinavir binding potency. Biochemistry.

[94] Liu Z., Yedidi R.S., Wang Y., Dewdney T.G., Reiter S.J., Brunzelle J.S., Kovari I.A., Kovari L.C. (2013). Insights into the mechanism of drug resistance: X-ray structure analysis of multi-drug resistant HIV-1 protease ritonavir complex. Biochem. Biophys. Res. Commun..

[95] Coman R.M., Robbins A.H., Fernandez M.A., Gilliland C.T., Sochet A.A., Goodenow M.M., McKenna R., Dunn B.M. (2008). The contribution of naturally occurring polymorphisms in altering the biochemical and structural characteristics of HIV-1 subtype C protease. Biochemistry.

[96] Tie Y., Wang Y.-F., Boross P.I., Chiu T.-Y., Ghosh A.K., Tozser J., Louis J.M., Harrison R.W., Weber I.T. (2012). Critical differences in HIV-1 and HIV-2 protease specificity for clinical inhibitors. Protein Sci..

[97] Stoll V., Qin W., Stewart K.D., Jakob C., Park C., Walter K., Simmer R.L., Helfrich R., Bussiere D., Kao J. (2002). X-ray crystallographic structure of ABT-378 (Lopinavir) bound to HIV-1 protease. Bioorganic Med. Chem..

[98] Klei H.E., Kish K., Lin P.-F.M., Guo Q., Friborg J., Rose R.E., Zhang Y., Goldfarb V., Langley D.R., Wittekind M. (2007). X-ray crystal structures of Human Immunodeficiency Virus Type 1 protease mutants complexed with Atazanavir. J. Virol..

[99] Wong-Sam A., Wang Y.-F., Zhang Y., Ghosh A.K., Harrison R.W., Weber I.T. (2018). Drug resistance mutation L76V alters nonpolar interactions at the flap–core interface of HIV-1 protease. ACS Omega.

[100] Kožíšek M., Lepšík M., Grantz Šašková K., Brynda J., Konvalinka J., Řezáčová P. (2014). Thermodynamic and structural analysis of HIV protease resistance to darunavir—Analysis of heavily mutated patient-derived HIV-1 proteases. FEBS J..

[101] Rawlings N.D., Barrett A.J., Thomas P.D., Huang X., Bateman A., Finn R.D. (2017). The MEROPS database of proteolytic enzymes, their substrates and inhibitors in 2017 and a comparison with peptidases in the PANTHER database. Nucleic Acids Res..

[102] Tamura K., Stecher G., Kumar S. (2021). MEGA11: Molecular Evolutionary Genetics Analysis Version 11. Mol. Biol. Evol..

[103] Thompson J.D., Higgins D.G., Gibson T.J. (1994). CLUSTAL W: Improving the sensitivity of progressive multiple sequence alignment through sequence weighting, position-specific gap penalties and weight matrix choice. Nucleic Acids Res..

[104] Subramanian B., Gao S., Lercher M.J., Hu S., Chen W.-H. (2019). Evolview v3: A webserver for visualization, annotation, and management of phylogenetic trees. Nucleic Acids Res..

[105] Zhou Z.-J., Qiu Y., Pu Y., Huang X., Ge X.-Y. (2020). BioAider: An efficient tool for viral genome analysis and its application in tracing SARS-CoV-2 transmission. Sustain. Cities Soc..

[106] Pei J., Kim B.H., Grishin N.V. (2008). PROMALS3D: A tool for multiple protein sequence and structure alignments. Nucleic Acids Res..

[107] Madhavi Sastry G., Adzhigirey M., Day T., Annabhimoju R., Sherman W. (2013). Protein and ligand preparation: Parameters, protocols, and influence on virtual screening enrichments. J. Comput.-Aided Mol. Des..

[108] Sharma R., Kesari P., Kumar P., Tomar S. (2018). Structure-function insights into chikungunya virus capsid protein: Small molecules targeting capsid hydrophobic pocket. Virology.

