# Supplementary material for: Computer-Aided Prediction of the Interactions of Viral Proteases with Antiviral Drugs: Antiviral Potential of Broad-Spectrum Drugs
Source: Molecules. 2023 Dec 31;29(1):225. doi: 10.3390/molecules29010225 (PMC10780089; doi:10.3390/molecules29010225)
Supplement: Supplementary file 1 [file molecules-29-00225-s001.zip › Supporting Information.pdf]

Supporting Information

Computer-Aided Prediction of the Interactions of Viral Proteases with Antiviral Drugs: Antiviral Potential of Broad-Spectrum Drugs

Pengxuan Ren [1], Shiwei Li [1], Shihang Wang [1], Xianglei Zhang [1,*] and Fang Bai [1,2,3,*]

[1] School of Life Science and Technology, Shanghai Institute for Advanced Immunochemical Studies, ShanghaiTech University, Shanghai 201210, China; renpx@shanghaitech.edu.cn (P.R.); lishw@shanghaitech.edu.cn (S.L.); wangshh12022@shanghaitech.edu.cn (S.W.)

[2] School of Information Science and Technology, ShanghaiTech University, Shanghai 201210, China

[3] Shanghai Clinical Research and Trial Center, Shanghai 201210, China

* Correspondence: zhangxl6@shanghaitech.edu.cn (X.Z.); baifang@shanghaitech.edu.cn (F.B.)

Table S1. The approved small molecule drugs targeting eleven viruses.

Types	Viruses	Targets	Small molecule drugs
dsDNA	HSV	DNA polymerase	Idoxuridine, Vidarabine, Trifluridine, Acyclovir, Foscarnet, Famciclovir, Valacyclovir, hydrochloride, Penciclovir, Brivudine, Acyclovir
	VZV	DNA polymerase	Vidarabine, Acyclovir, Brivudine
	HCMV	DNA polymerase	Ganciclovir, Foscarnet, Cidofovir, Valganciclovir
		DNA terminase complex	Letermovir
	VARV	DNA polymerase	Brincidofovir
		VP37	Tecovirimat
ssDNA	--	--	--
dsDNA (RT)	HBV	DNA polymerase	Lamivudine, Adefovir dipivoxil, Entecavir, Telbivudine, Tenofovir disoproxil fumarate, Tenofovir alafenamide fumarate
dsRNA	--	--	--
(+) ssRNA	HCV	NS3/4A protease	Boceprevir, Telaprevir, Simeprevir, Paritaprevir, Asunaprevir, Vaniprevir, Grazoprevir, Voxilaprevir, Glecaprevir, Paritaprevir
		NS5B polymerase	Sofosbuvir, Dasabuvir sodium
		NS5A protein	Pibrentasvir, Velpatasvir, Elbasvir, Daclatasvir, Ombitasvir, Ledipasvir
	SARS-CoV-2	RNA polymerase	Remdesivir, Molnupiravir, Azvudine, Deuremidevir Hydrobromide
		$3CL^{pro}$ protease	Nirmatrelvir, Ensitrelvir, SIM0417, RAY1216
(-) ssRNA	FluV	M2 channel protein	Amantadine, Rimantadine
		RNA polymerase	Ribavirin, Favipiravir
		Neuraminidase	Zanamivir, Oseltamivir, Laninamivir octanoate, Peramivir
		Endonuclease	Baloxavir marboxil
	RSV	RNA polymerase	Ribavirin
	HDV	NTCP (Host target)	Bulevirtide
ssRNA (RT)	HIV	Reverse transcriptase	Zidovudine, Didanosine, Zalcitabine, Stavudine, Lamivudine, Nevirapine, Delavirdine mesylate, Efavirenz, Abacavir sulfate, Tenofovir disoproxil fumarate, Emtricitabine, Etravirine, Tenofovir alafenamide fumarate, Rilpivirine, Doravirine
		Protease	Saquinavir mesylate, Indinavir sulfate, Ritonavir, Nelfinavir mesylate, Amprenavir, Lopinavir, Fosamprenavir calcium, Atazanavir sulfate, Tipranavir, Darunavir
		GP41 glycoprotein	Enfuvirtide
		Integrase	Elvitegravir, Raltegravir, Dolutegravir, Bictegravir
		Host CCR5 receptor	Maraviroc

Figure S1. The PROMALS3D alignment of viral 3CL^{pro}.

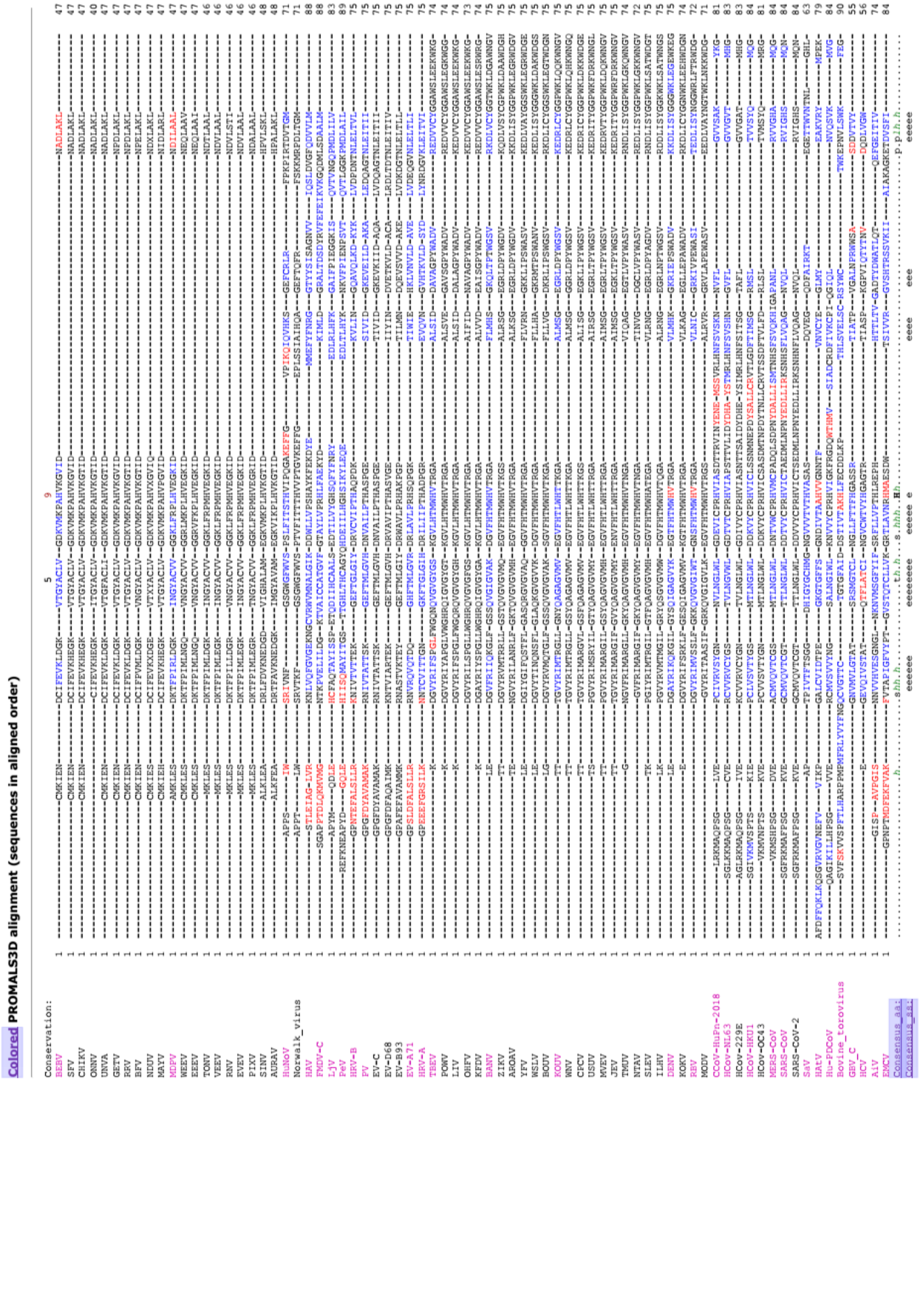

Figure S1. The PROMALS3D alignment of viral 3CLpro.

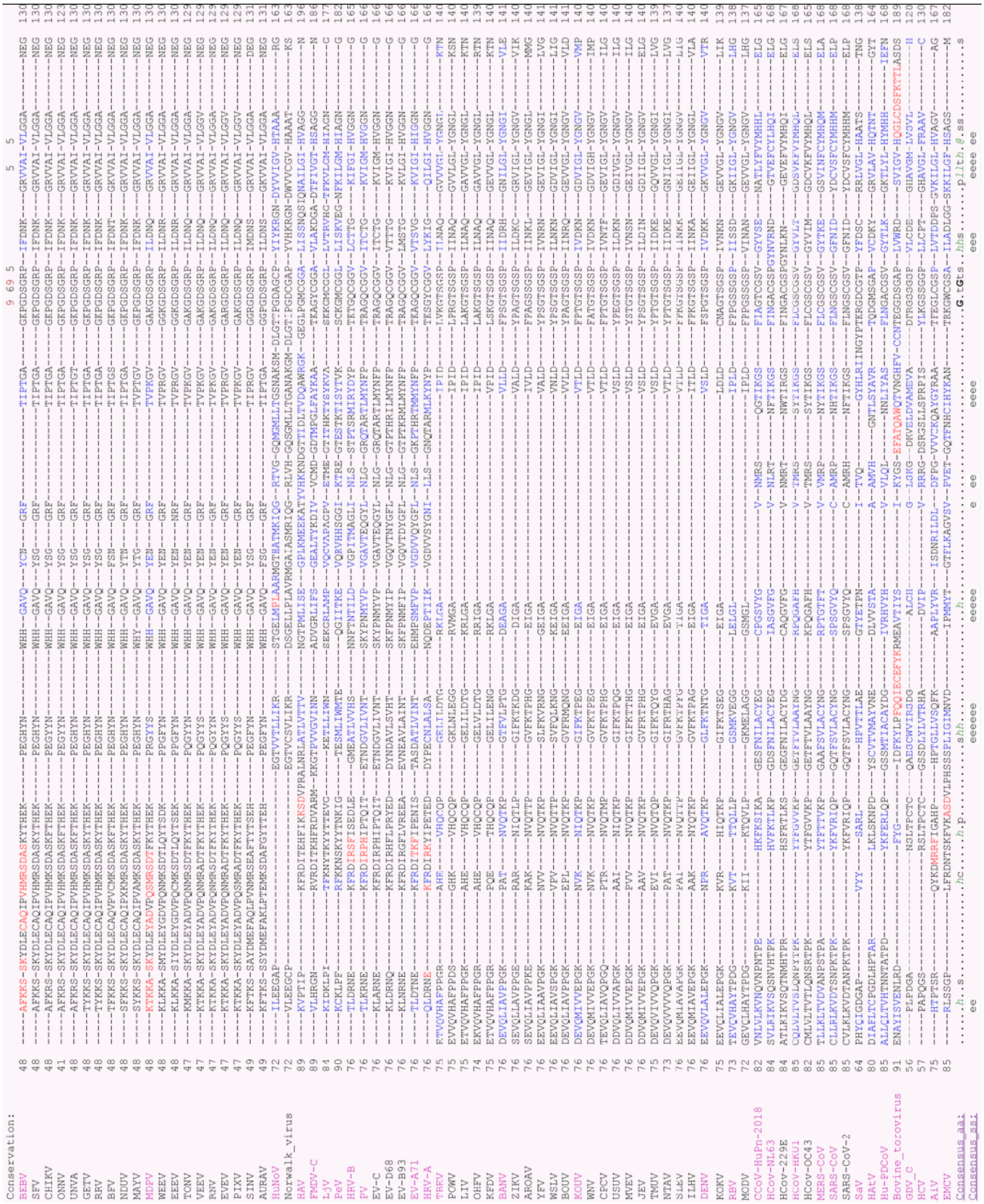

Figure S1. The PROMALS3D alignment of viral 3CLpro.

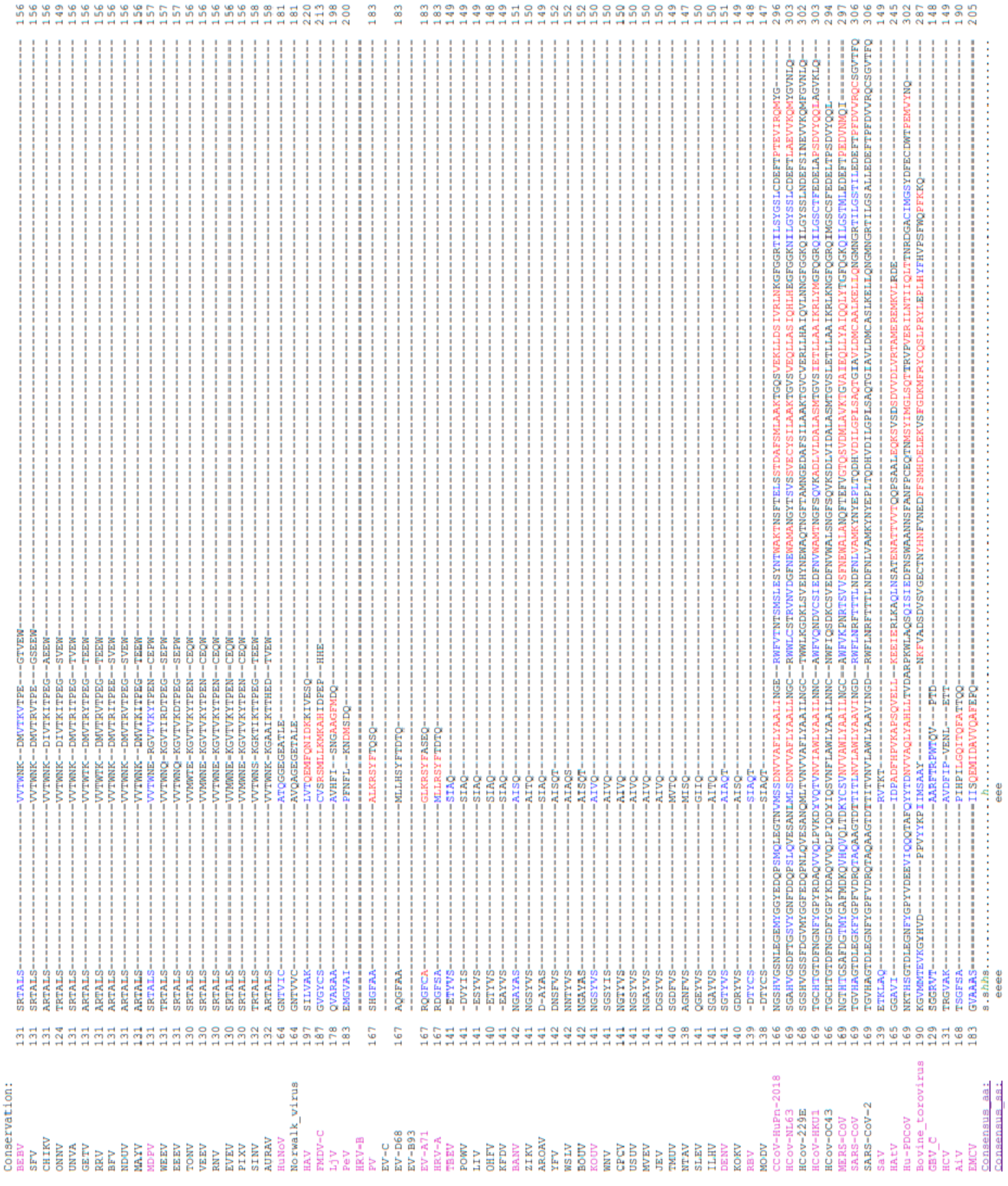

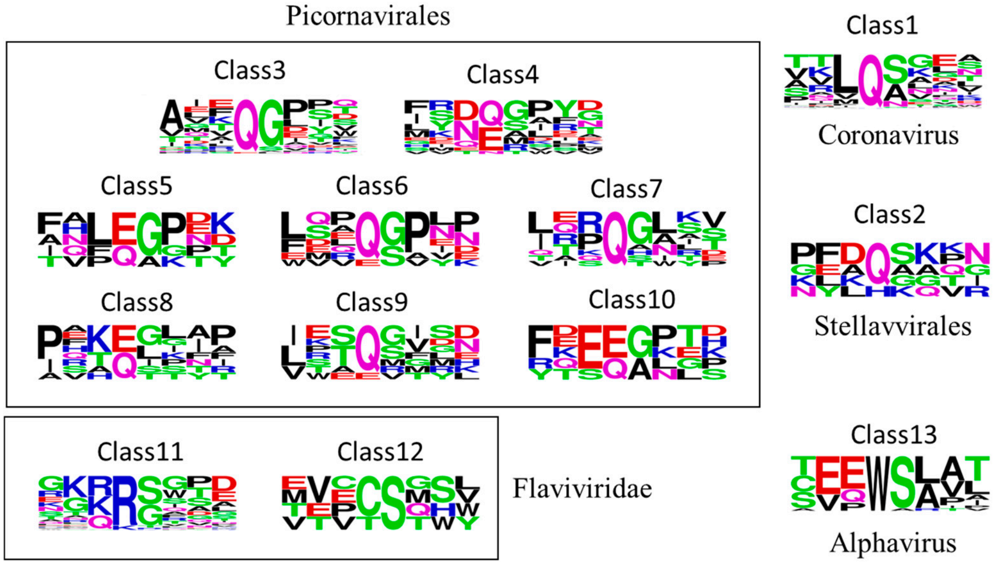

Figure S2. The substrate sequences of 13 classes of viruses 3CL^{pro}.

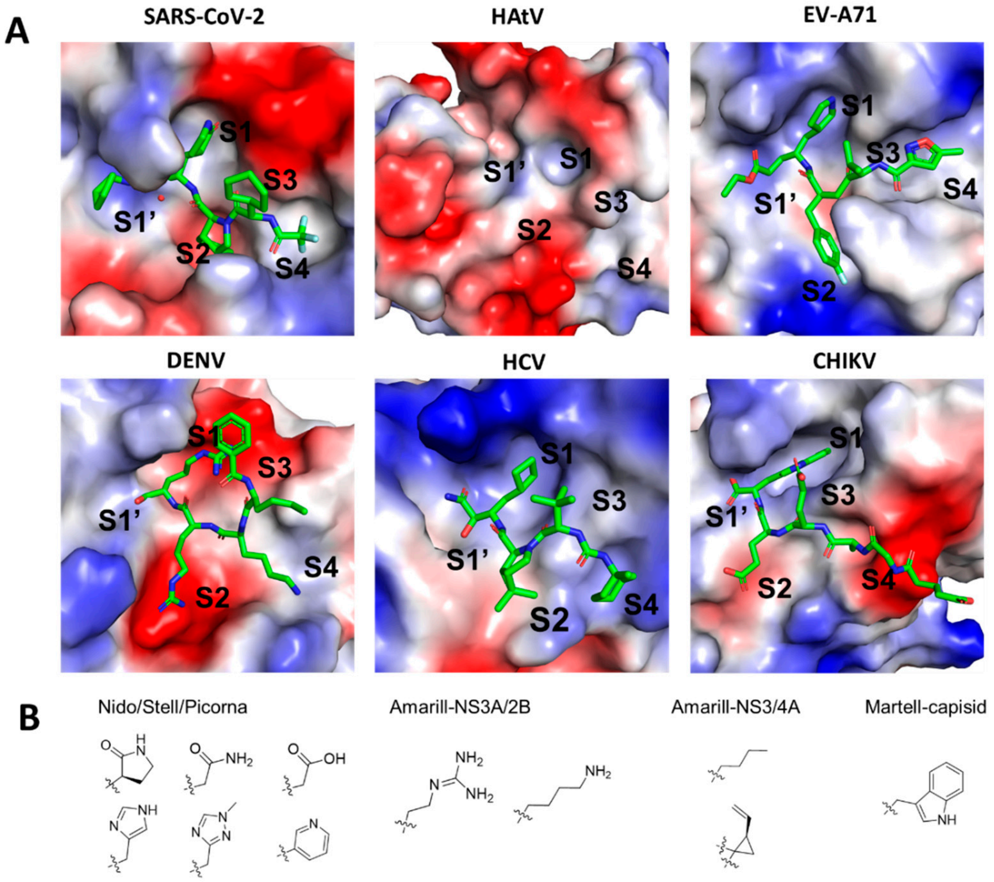

Figure S3. The active sites comparison of viral 3CL^{pro}. (A) The surface of the electrostatic potential of SARS-CoV-2, HAtV, EV-A71, DENV, HCV, and CHIKV. The PDB codes are 8IGN, 2W5E, 3SJO, 3U1I, 3LOX, and 5H23, respectively. The substrates or inhibitors are shown as sticks. (B) P1 groups of S1 sites.

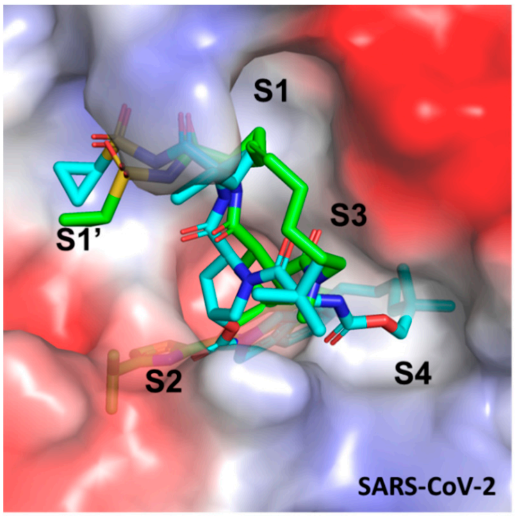

Figure S4. The predicted binding modes of SARS-CoV-2 3CL^{pro} with Vaniprevir or Simeprevir. Proteins are shown as the surface of the electrostatic potential. Vaniprevir (cyan) and Simeprevir (green) are presented as sticks.

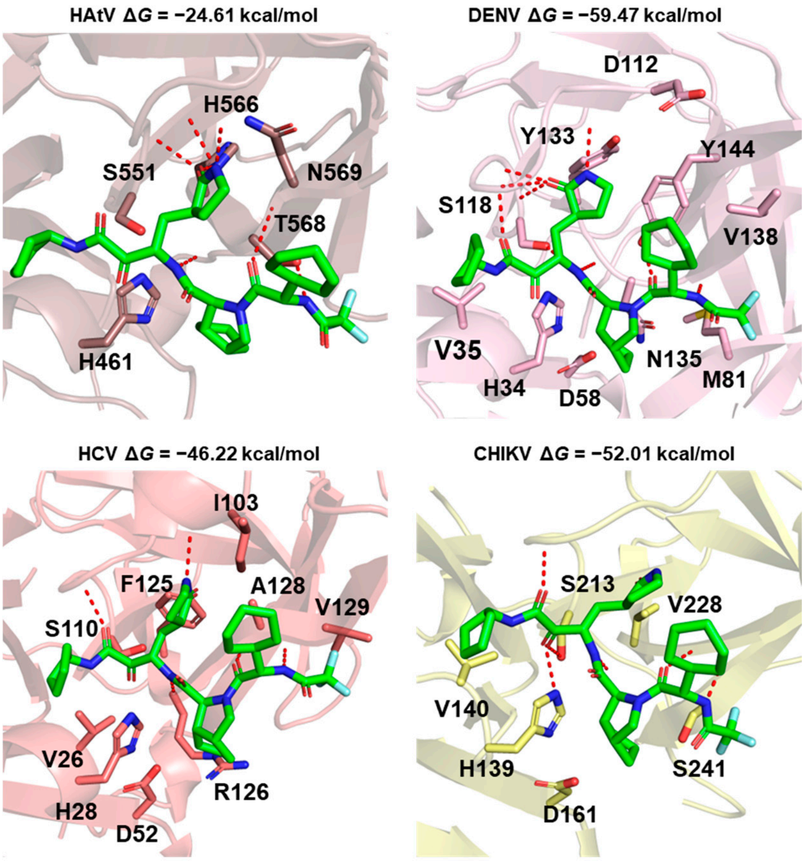

Figure S5. Predicted binding modes of HAtV, DENV, HCV, and CHIKV 3CL^{pro} with Ray1216. The proteins are shown as cartoon models. The Ray1216 and interaction residues are depicted as sticks. The red dashed lines are hydrogen bonds between the ligands and proteins. HAtV (PDB code: 2W5E) and CHIKV (PDB code: 5H23) 3CL^{pro} are retrieved from PDB database [1,2]. The other protein structures were predicted by ColabFold.

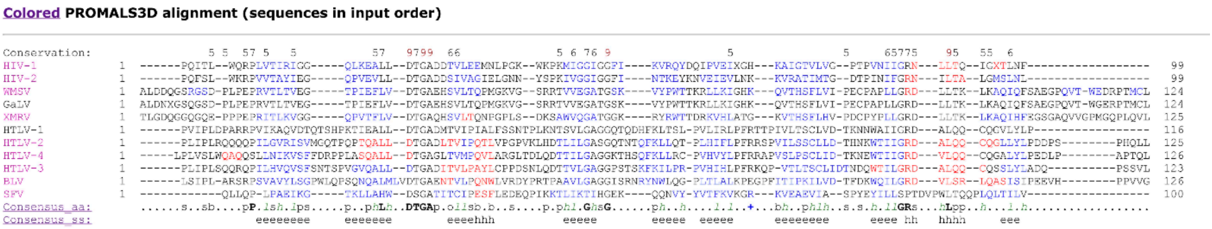

Figure S6. The PROMALS3D alignment of viral PAPs.

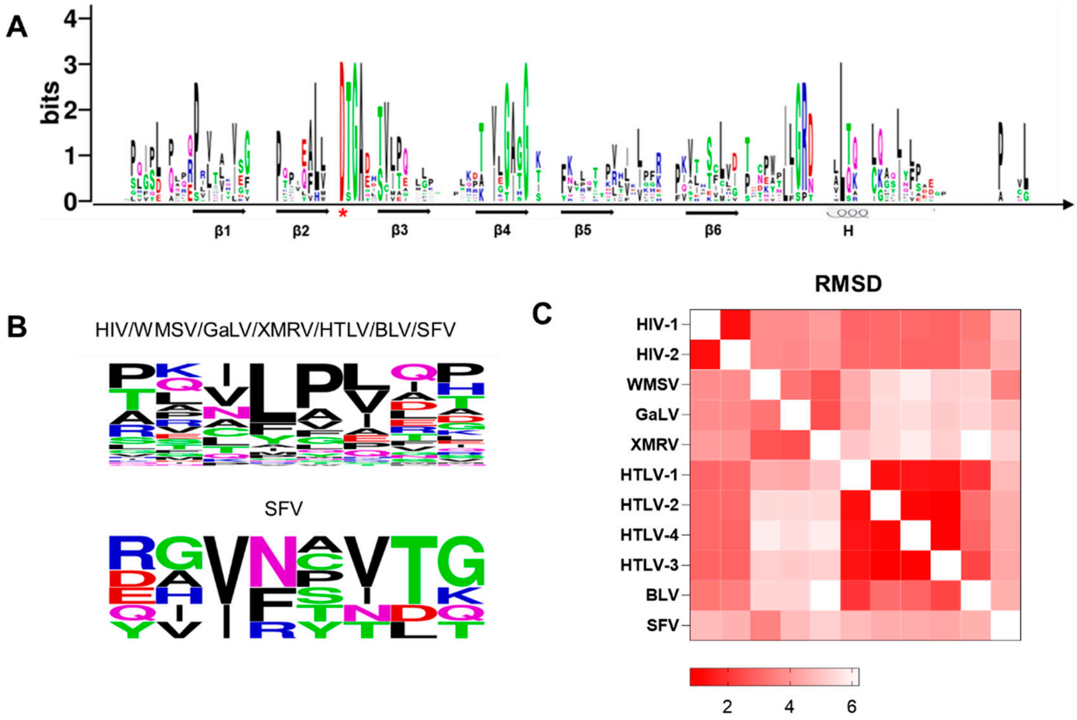

Figure S7. The sequences, substrates, and RMSD values of viral PAPs. (A) Sequence logo of multiple sequence alignment. Arrows represent β-sheets. Asterisks represent catalytic residues. The spiral chart represents α-helix. (B) Sequence logo of PAPs cleavage sites. Residues are scaled according to their frequencies at each position. (C) The RMSD heat map of PAPs.

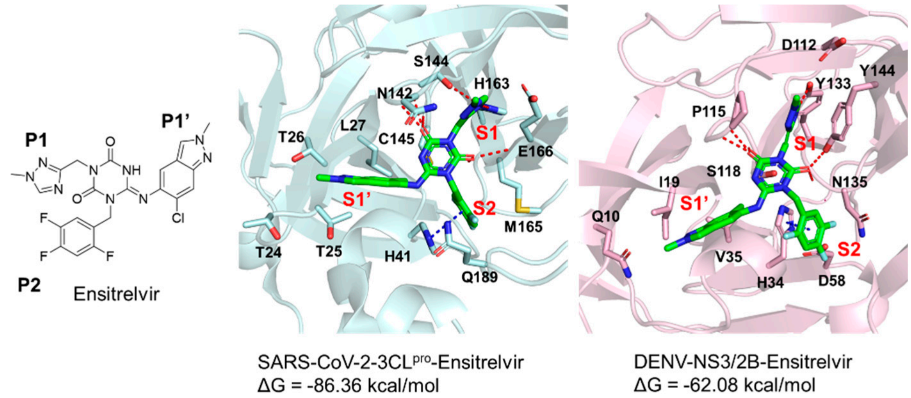

Figure S8. The complex structures of SARS-CoV-2 3CL^{pro}-Ensitrelvir and DENV NS3/2B Ensitrelvir. The PDB code of SARS-CoV-2 3CL^{pro}-Ensitrelvir is 7VU6 [3]. The structure of DENV NS3/2B is predicted by ColabFold [4].

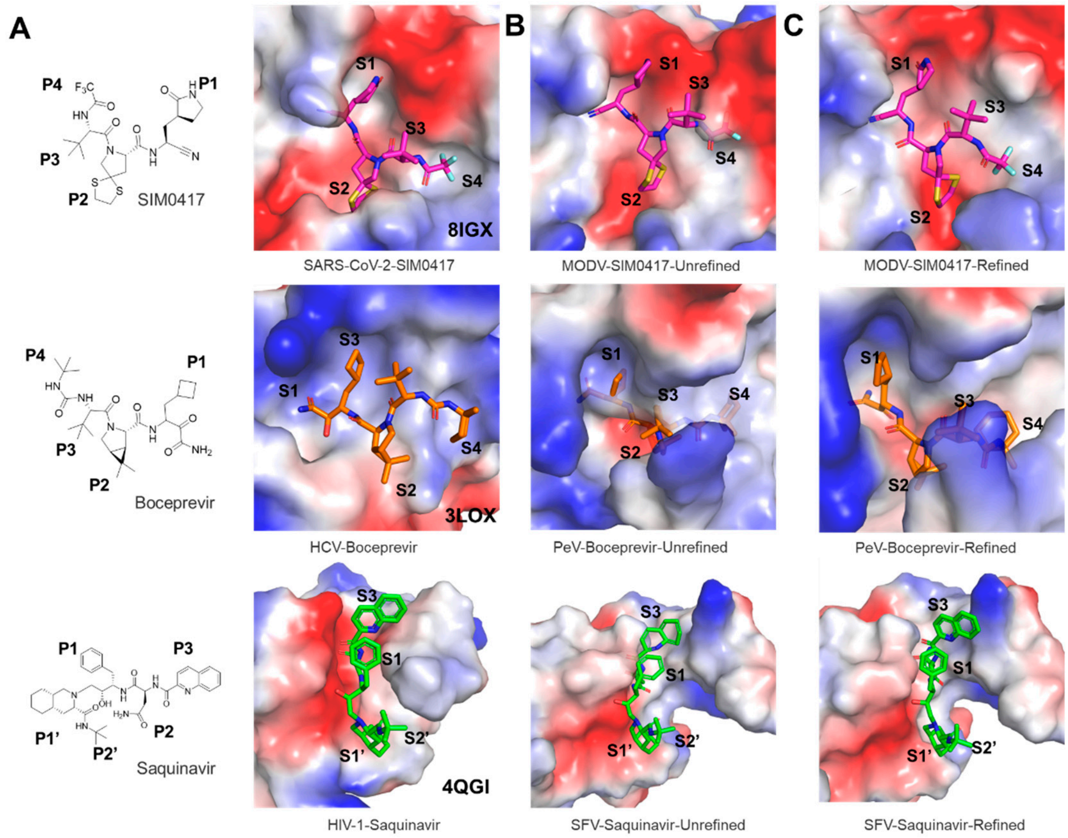

Figure S9. The complex structures of viral proteases with protease inhibitors. (A) The 2D structures of SIM0417, Boceprevir, and Saquinavir, and the complex structures of SARS-CoV-2 3CL^{pro}-SIM0417 (PDB code: 8IGX), HCV NS3/4A-Boceprevir (PDB code: 3LOX), and HIV-1 Protease-Saquinavir (PDB code: 4QGI) [5-7]. (B) The unrefined complex structures of MODV NS3/2B-SIM0417, PeV 3CL^{pro}-Boceprevir, and SFV Protease-Saquinavir. (C) The refined complex structures of MODV NS3/2B-SIM0417, PeV 3CL^{pro}-Boceprevir, and SFV Protease-Saquinavir. The other protein structures are predicted by ColabFold [4].

Reference

1. Speroni, S.; Rohayem, J.; Nenci, S.; Bonivento, D.; Robel, I.; Barthel, J.; Luzhkov, V.B.; Coutard, B.; Canard, B.; Mattevi, A. Structural and biochemical analysis of human pathogenic Astrovirus serine protease at 2.0 Å resolution. *Journal of Molecular Biology* **2009**, *387*, 1137-1152, doi:https://doi.org/10.1016/j.jmb.2009.02.044.
2. Sharma, R.; Kesari, P.; Kumar, P.; Tomar, S. Structure-function insights into chikungunya virus capsid protein: small molecules targeting capsid hydrophobic pocket. *Virology* **2018**, *515*, 223-234, doi:https://doi.org/10.1016/j.virol.2017.12.020.
3. Unoh, Y.; Uehara, S.; Nakahara, K.; Nobori, H.; Yamatsu, Y.; Yamamoto, S.; Maruyama, Y.; Taoda, Y.; Kasamatsu, K.; Suto, T.; et al. Discovery of S-217622, a noncovalent oral SARS-CoV-2 3CL protease inhibitor clinical candidate for treating COVID-19. *Journal of Medicinal Chemistry* **2022**, *65*, 6499-6512.
4. Mirdita, M.; Schütze, K.; Moriwaki, Y.; Heo, L.; Ovchinnikov, S.; Steinegger, M. ColabFold: making protein folding accessible to all. *Nature Methods* **2022**, *19*, 679-682, doi:10.1038/s41592-022-01488-1.
5. Jiang, X.; Su, H.; Shang, W.; Zhou, F.; Zhang, Y.; Zhao, W.; Zhang, Q.; Xie, H.; Jiang, L.; Nie, T.; et al. Structure-based development and preclinical evaluation of the SARS-CoV-2 3C-like protease inhibitor simnotrelvir. *Nature Communications* **2023**, *14*, 6463, doi:10.1038/s41467-023-42102-y.
6. Bennett, F.; Huang, Y.; Hendrata, S.; Lovey, R.; Bogen, S.L.; Pan, W.; Guo, Z.; Prongay, A.; Chen, K.X.; Arasappan, A.; et al. The introduction of P4 substituted 1-methylcyclohexyl groups into Boceprevir®: A change in direction in the search for a second generation HCV NS3 protease inhibitor. *Bioorganic & Medicinal Chemistry Letters* **2010**, *20*, 2617-2621, doi:https://doi.org/10.1016/j.bmcl.2010.02.063.
7. Goldfarb, N.E.; Ohanessian, M.; Biswas, S.; McGee, T.D., Jr.; Mahon, B.P.; Ostrov, D.A.; Garcia, J.; Tang, Y.; McKenna, R.; Roitberg, A.; et al. Defective hydrophobic sliding mechanism and active site expansion in HIV-1 protease drug resistant variant Gly48Thr/Leu89Met: mechanisms for the loss of saquinavir binding potency. *Biochemistry* **2015**, *54*, 422-433, doi:10.1021/bi501088e.
